# Genomic Analysis of Waterpipe Smoke-Induced Lung Tumor Autophagy and Plasticity

**DOI:** 10.3390/ijms23126848

**Published:** 2022-06-20

**Authors:** Rania Faouzi Zaarour, Mohak Sharda, Bilal Azakir, Goutham Hassan Venkatesh, Raefa Abou Khouzam, Ayesha Rifath, Zohra Nausheen Nizami, Fatima Abdullah, Fatin Mohammad, Hajar Karaali, Husam Nawafleh, Yehya Elsayed, Salem Chouaib

**Affiliations:** 1 Thumbay Research Institute for Precision Medicine, Gulf Medical University, Ajman 4184, United Arab Emirates; dr.rania@gmu.ac.ae (R.F.Z.); gouthamhv@gmail.com (G.H.V.); dr.raefa@gmu.ac.ae (R.A.K.); ayesha@gmu.ac.ae (A.R.); zohranausheennizami@gmail.com (Z.N.N.); 2017bm10@mygmu.ac.ae (F.A.); 2017bm07@mygmu.ac.ae (F.M.); husam@gmu.ac.ae (H.N.); 2 National Center for Biological Sciences, Tata Institute of Fundamental Research, Bangalore 560065, India; mohaks@ncbs.res.in; 3 School of Life Science, The University of Trans-Disciplinary Health Sciences & Technology (TDU), Bangalore 560064, India; 4 Molecular and Translational Medicine Laboratory, Faculty of Medicine, Beirut Arab University, Beirut 11072809, Lebanon; b.azakir@bau.edu.lb (B.A.); hajar.karaali@hotmail.com (H.K.); 5 Department of Biology, Chemistry and Environmental Sciences (BCE), American University of Sharjah, Sharjah 26666, United Arab Emirates; yehyaelsayed@gmail.com; 6 Inserm Umr 1186, Integrative Tumor Immunology and Immunotherapy, Gustave Roussy, Faculty of Medicine, University Paris-Saclay, 94805 Villejuif, France

**Keywords:** autophagy, tumor mutational burden, tumor microenvironment, waterpipe smoke, lung cancer

## Abstract

The role of autophagy in lung cancer cells exposed to waterpipe smoke (WPS) is not known. Because of the important role of autophagy in tumor resistance and progression, we investigated its relationship with WP smoking. We first showed that WPS activated autophagy, as reflected by LC3 processing, in lung cancer cell lines. The autophagy response in smokers with lung adenocarcinoma, as compared to non-smokers with lung adenocarcinoma, was investigated further using the TCGA lung adenocarcinoma bulk RNA-seq dataset with the available patient metadata on smoking status. The results, based on a machine learning classification model using Random Forest, indicate that smokers have an increase in autophagy-activating genes. Comparative analysis of lung adenocarcinoma molecular signatures in affected patients with a long-term active exposure to smoke compared to non-smoker patients indicates a higher tumor mutational burden, a higher CD8+ T-cell level and a lower dysfunction level in smokers. While the expression of the checkpoint genes tested—PD-1, PD-L1, PD-L2 and CTLA-4—remains unchanged between smokers and non-smokers, B7-1, B7-2, IDO1 and CD200R1 were found to be higher in non-smokers than smokers. Because multiple factors in the tumor microenvironment dictate the success of immunotherapy, in addition to the expression of immune checkpoint genes, our analysis explains why patients who are smokers with lung adenocarcinoma respond better to immunotherapy, even though there are no relative differences in immune checkpoint genes in the two groups. Therefore, targeting autophagy in lung adenocarcinoma patients, in combination with checkpoint inhibitor-targeted therapies or chemotherapy, should be considered in smoker patients with lung adenocarcinoma.

## 1. Introduction

Lung cancer is the second most common diagnosed type of cancer in men and women, after prostate and breast cancers, respectively [1]. The greatest number of deaths are due to cancers of the lung, which account for 25% of all cancer-related deaths [1]. Tobacco smoking is the most common cause for lung cancer [2]. One type of tobacco smoking is waterpipe smoking (WPS), where the smoke of the tobacco passes through water prior to being inhaled. WP use is on the rise globally [3], and there is a strong link between WPS and lung cancer [4,5]. Because of the toxicants present in WPS, smokers are exposed to a large amount and variety of chemicals, including many carcinogens [6,7]. WPS has been shown to result in the generation of free radicals, reactive oxygen species (ROS) and inflammation [8,9,10].

Previous studies have shown that WPS condensate (WPSC) treatment of lung cancer cell lines modulates cell plasticity. WPSC induced epithelial to mesenchymal transition (EMT), cancer stem cell (CSC) features, and an increase in inflammation and DNA damage [11,12]. The consequences of DNA damage depend on the cell type and on the extent and intensity of the stress and could activate senescence, autophagy, or cell death programs. Apoptosis functions to suppress tumor growth, while autophagy can be activated in different cells at different stages of tumor growth and has paradoxical roles as it can suppress or promote tumor growth depending on the type and stage of the tumor [13]. While apoptosis fulfills its role through dismantling damaged or unwanted cells, autophagy maintains cellular homeostasis through recycling selective intracellular organelles and molecules. Autophagy is activated by different metabolic stressors in the tumor microenvironment (TME), including hypoxia, nutrient deprivation, and inflammation. In the context of WPS, nicotine present in WPS and in cigarette smoke has been shown to induce bronchial epithelial cell apoptosis, senescence, and autophagy impairment in normal lung epithelial cells post treatment for up to 6 h [14,15,16].

The molecular switch between cell death and cell survival is a key determinant of cell fate and cancer progression. Tumor mutational burden (TMB) rises because of DNA damage response and repair gene alterations, which have direct implications on the immune cells’ landscape. An increase in TMB is associated with a favorable response to immune checkpoint inhibitors (ICI) [17] as this can increase immunogenic neoantigen production and its subsequent presentation by antigen-presenting cells, such as dendritic cells (DCs), to CD8+ T-cells, thus promoting their anticancer activity [18]. ICI have been increasingly used in the treatment of non-small cell lung cancer (NSCLC), enhancing response rates and long-term survival but only in a fraction of treated patients [19,20]. The most used ICI-based therapy is anti-PD-1 or anti-PD-L1, which work to block the inhibitory signaling between PD-1, present on the surface of activated T cells, and its ligand PD-L1, expressed on tumor cells [21]. The aim is to revitalize the immune response and eliminate tumor cells. Currently, the application of ICI in NSCLC is determined based on high microsatellite instability (MSI), TMB, PD-L1 expression, and disease burden [20]. These determinants are clearly insufficient to ensure patient response, and other factors in the TME could additionally be involved. Indeed, the TME is a collection of cellular components, including tumor, immune, and endothelial cells, as well as non-cellular components, such as extracellular matrix and signaling factors, cytokines, and chemokines, all of which are functioning together in acidic, hypoxic and nutrient-deprived conditions [22]. Tumor-promoting immune cells, such as myeloid-derived suppressor cells (MDSCs), M2 macrophages and regulatory T cells (Tregs), tend to thrive in such an environment, while tumor antagonizing-cells, including CD8+ T cells and natural killer (NK) cells, tend to be inhibited or even excluded from the tumor site [22]. A better understanding of how these features merge in lung adenocarcinoma patients exposed to smoke is needed to better delineate their response rates following immunotherapy.

Our study addresses the role of WPS on autophagy, on TMB in lung cancer cell lines and using TCGA datasets of lung adenocarcinoma patients with a history of smoking. We further investigated the immunological landscape in these datasets. In vitro, we observed an increase in apoptosis at early exposure times followed by an activation of autophagy at longer treatment duration. Long-term exposure up to 6 months in lung cancer cell lines identified an increase in TMB that was also depicted in our analysis of TCGA datasets. Further analysis of the immune landscape of lung adenocarcinoma patients identified no change in immune checkpoint inhibitors between smokers and non-smokers. We also observed an increase in NK cells and CD8+ T cells, coupled by lower T-cell dysfunction. However, there were lower dendritic cell numbers. The current studies point to autophagy as a potential target for treatment of lung adenocarcinoma patients with a history of smoking. Our results are suggestive of better prognosis of smokers with lung adenocarcinoma post immunotherapy treatment.

## 2. Results

### 2.1. Waterpipe Smoke Condensate Increases Apoptosis and Activates Autophagy in Lung Cancer Cell Lines

**Figure 1:**
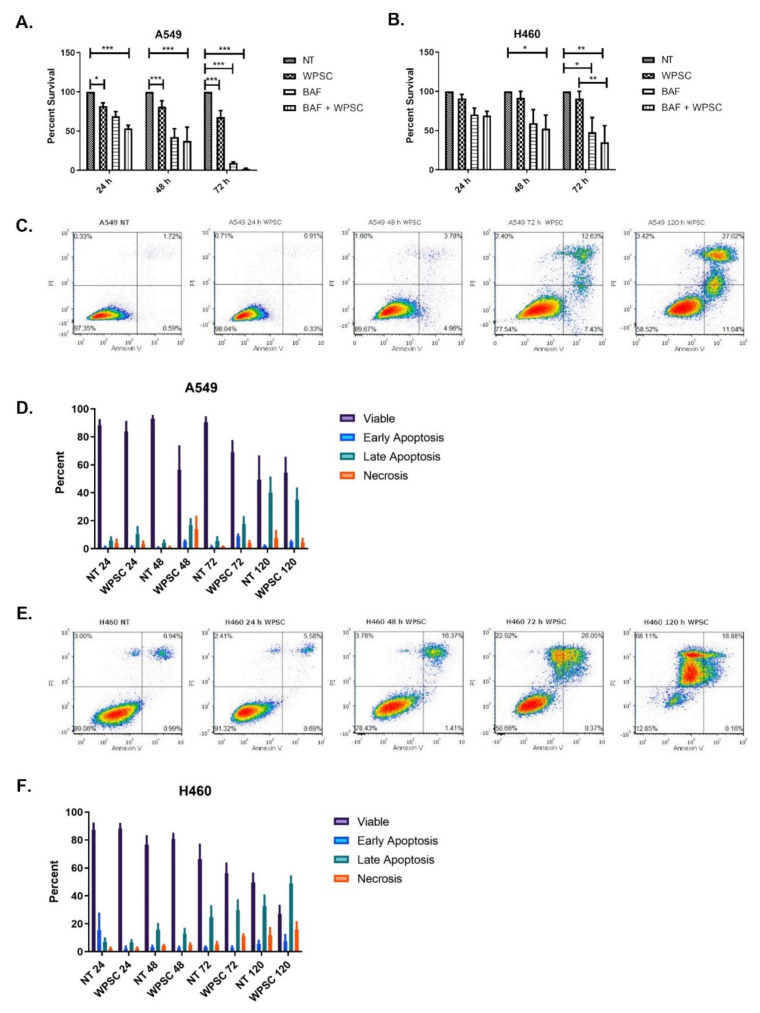
WPSC increases apoptosis and autophagy in lung cancer cell lines. Cell viability in response to 0.5% WPSC was measured using MTT assay in A549 (**A**) and H460 (**B**) cell lines at 24, 48 and 72 h. Apoptosis was measured by flow cytometry. Cells were stained with a combination of Annexin V-FITC, propidium iodide (PI) following WPSC treatment, in A549 (**C**,**D**) and H460 (**E**,**F**). Results represent means of three independent experiments, and data represent mean ± standard error of mean. * *p* ≤ 0.05, ** *p* ≤ 0.01 and *** *p* ≤ 0.001.

We first investigated the cytotoxic effects of waterpipe smoke condensate (WPSC) and its impact on autophagy. For this purpose, both A549 and H460 lung cancer cell lines were treated with 0.5% WPSC. This WPSC concentration was previously found to cause only a small fraction of A549 and H460 cells to die [11]. Cell viability using the MTT assay at 24, 48 and 72 h was measured. As depicted in Figure 1A,B, A549 cells displayed reduced viability in response to WPSC, whereas H460 cells did not up till 72 h of treatment. The vacuolar (H+) ATPase (V-ATPase) inhibitor Bafilomycin A1 (BafA1) was used to inhibit autophagy [23]. We observed a decrease in cell viability in response to 100 nM of BafA1 in both cell lines. The concomitant treatment of BafA1 and WPSC resulted in an additive negative effect on cell viability that was significant at 72 h, indicating that autophagy pathways could be contributing to cell survival following WPSC treatment.

Autophagy and apoptosis are both important in maintaining cellular homeostasis. Stress-inducing signals influence both apoptosis and autophagy, and while functionally distinct, a crosstalk between the two could play an important role in pathological processes, including cancer. As we observed a decrease in cell viability following WPSC treatment, we asked whether apoptosis was activated. Treating A549 and H460 cells with 0.5% WPSC up to 5 days (120 h) resulted in a decrease in cell viability with a gradual increase in apoptosis as measured by an increase in Annexin V/PI positive cells (Figure 1C–F).

**Figure 2:**
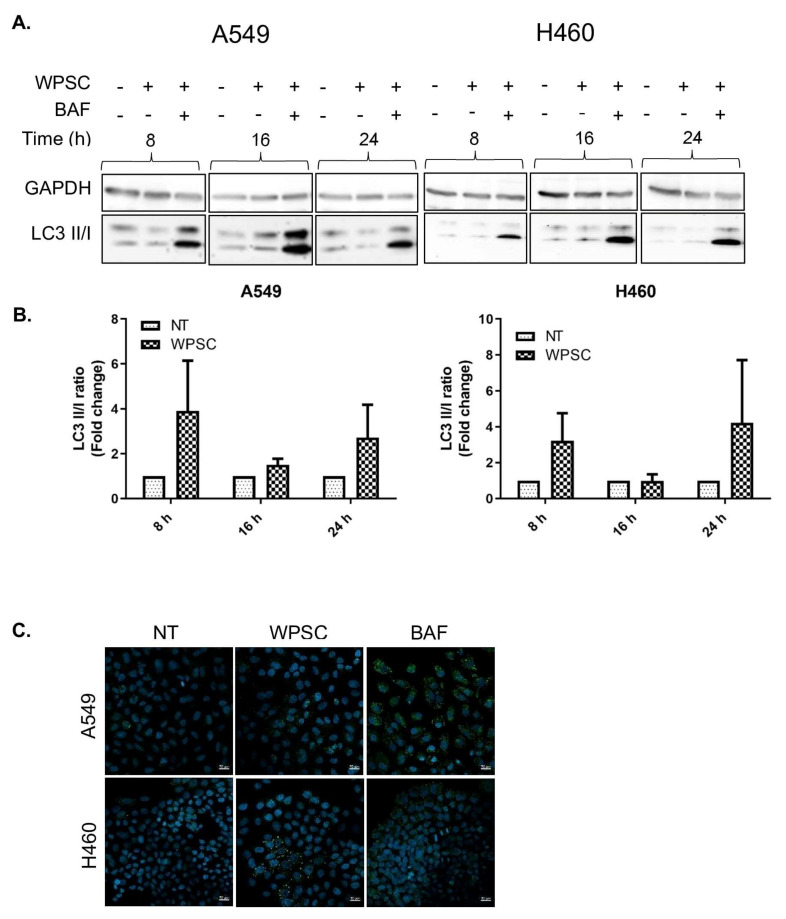

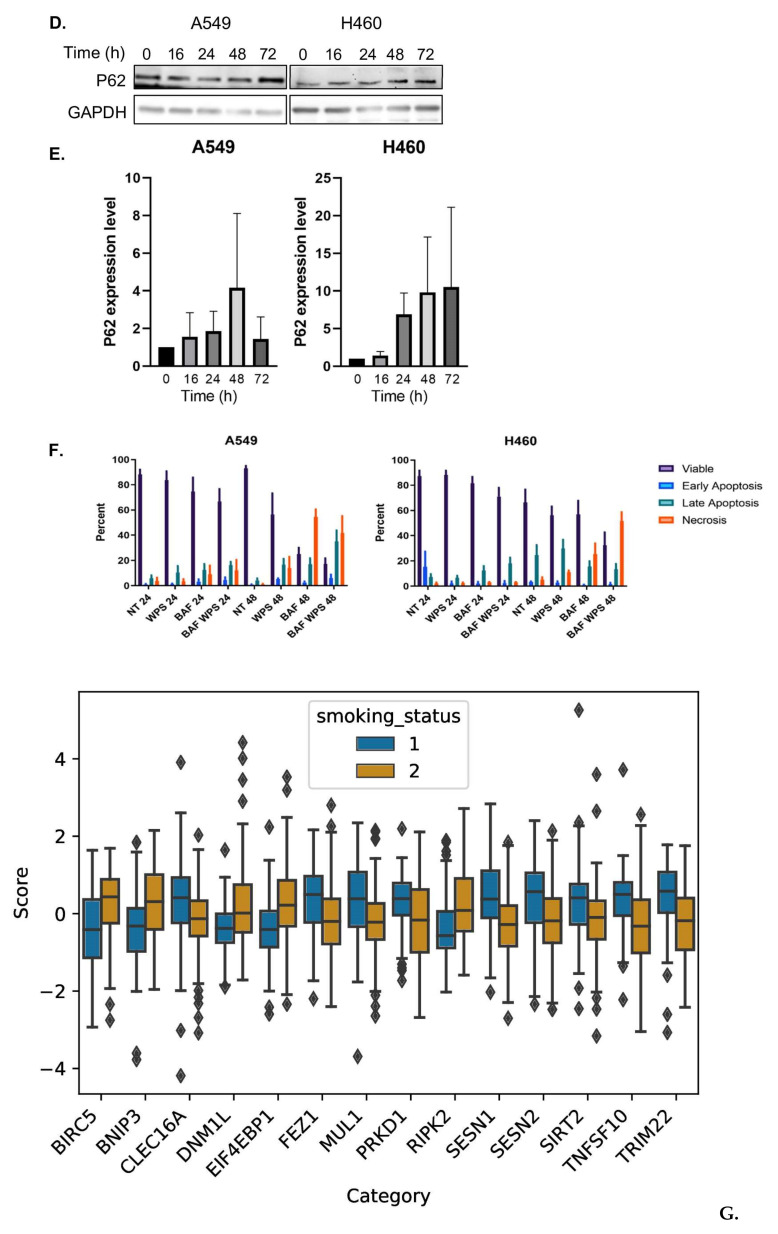

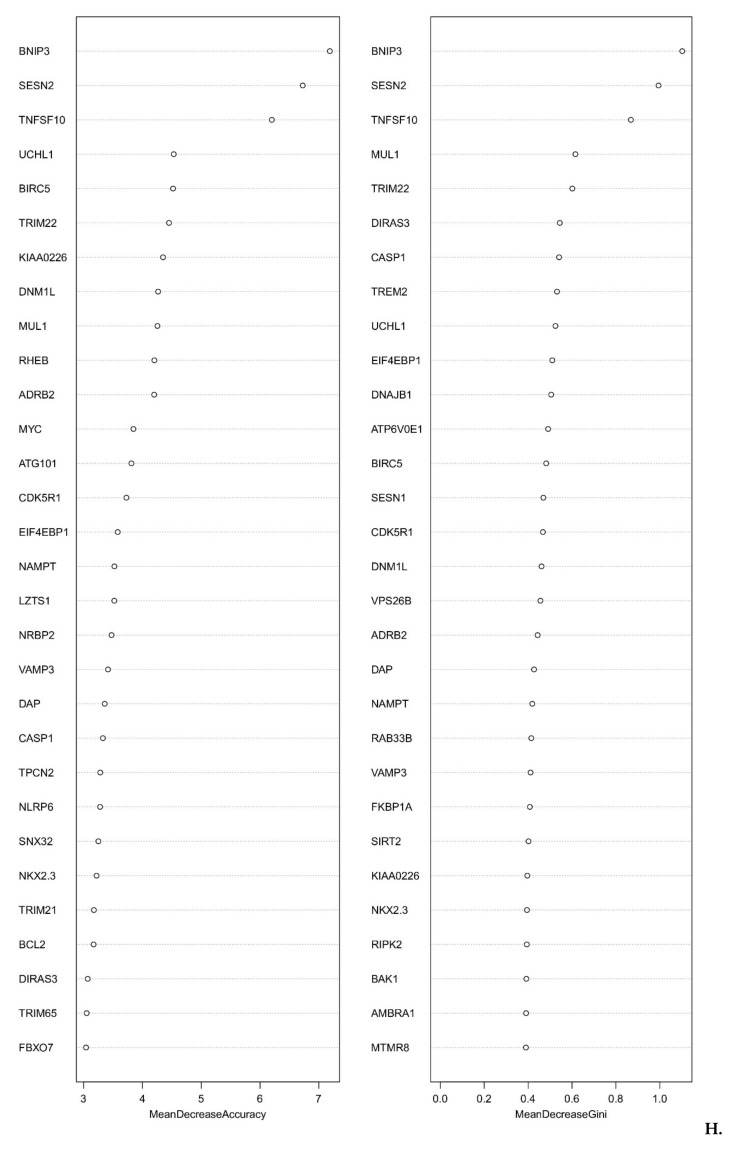
WPSC induces autophagy in lung cancer cell lines. A549 and H460 cell lines were treated with 0.5% WPSC for 24 h. LC3I/II levels were monitored by western blotting using standard procedures with anti-LC3 and GAPDH as a loading control for (**A**) band intensity was quantified in (**B**). The immunofluorescence analysis of p62 protein was performed following 72 h WPSC treatment; cells were treated with 100 nM Baf-A1 for 24 h as positive control (**C**). Western blotting for p62 protein was performed by standard procedures with anti-p62, and anti-GAPDH as a loading control (**D**) band intensity was quantified in (**E**). Cells were stained with a combination of Annexin V-FITC and propidium iodide (PI) to measure apoptosis, following 100nM Baf-A1 pre-treatment and WPSC treatment for the indicated time points, in both cell lines (**F**). TCGA lung adenocarcinoma bulk RNA-seq datasets of all autophagy genes between smokers and non-smokers (**G**). Two feature importance techniques were used—meanDecreaseAccuracy and meanDecreaseGini—to classify the top predictors of the autophagy-related genes with smoking status (**H**). Representative images of confocal microscopic analysis of p62 (green) and DAPI (blue) are shown. Scale bar, 10 µm. Results represent means of three independent experiments, and data represent mean ± standard error of mean.

Despite the increase in apoptotic cells, a large percentage of the cells survived the WPSC treatment; up to 60% of A549 and 30% of H460 cells remained viable following 5-day exposure. We therefore examined whether autophagy was activated following WPSC treatment. One method for detecting autophagic flux is by measuring differences in the amounts of LC3-II in the presence of an autophagy inhibitor; we thus analyzed the increase in the ratio of LC3-II to LC3-I by western blot with and without BafA1. The amount of LC3-II in WPSC-treated cells increased further in the presence of BafA1, which indicates an enhancement of autophagic flux starting at 8 h and up to 24 h (Figure 2A,B). The ubiquitin-associated protein p62, which binds to LC3, is also used to monitor autophagic flux; as such, we analyzed the expression levels of p62 following WPSC treatment. Immunofluorescence indicated an increase in p62 puncta, and western blots demonstrated an increase in p62 levels (Figure 2B). Because autophagy could promote cell survival, we analyzed whether WPSC in combination with autophagy inhibitors would result in a further increase in cell death. Pretreating the cells with BafA1 prior to WPSC exposure in A549 cells resulted in a slight increase in late apoptotic cells at 48 h when compared to BafA1-alone-treated cells. In H460 cells, the number of late apoptotic cells increased at 24 h, and necrotic cell death was more prominent at 48 h (Figure 2F). This result indicates that both cell lines are susceptible to stress-induced cell death, and that autophagy is important in maintaining the surviving cells. Therefore, manipulating pathways of apoptosis, necrosis and autophagy in cancer cells could skew cell fate decisions. We next sought to investigate if this autophagy response is specific to smokers with lung adenocarcinoma, as compared to non-smokers with lung adenocarcinoma. We analyzed the TCGA lung adenocarcinoma bulk RNA-seq dataset with the available patient metadata on smoking status. Using random-forest-based multivariate modeling implemented in GeneSrF, we obtained the top 14 autophagy genes as the best predictors of smoking status [24]. We compared the fold change in expression of all autophagy genes between smokers and non-smokers (Figure 2G). We also implemented our own random forest modeling using the randomForest package in R (model accuracy = 0.65, sensitivity = 0.96, and precision = 0.60; see methods). Using two feature importance techniques, meanDecreaseAccuracy and meanDecreaseGini, we found that there were four genes that were consistently reported as the top predictors of smoking status (Figure 2H). The results showed an activation of autophagy in smokers, and among the differentially expressed genes, BNIP3 (Wilcoxon rank sum test, *p*-value = 2.16 × 10^−5^) was significantly up-regulated in smokers, and SESN2 (Wilcoxon rank sum test, *p*-value = 1.67 × 10^−5^), TRIM22 (Wilcoxon rank sum test, *p*-value = 2.9 × 10^−7^) and TNFSF10 (Wilcoxon rank sum test, *p*-value = 1.74 × 10^−6^) were significantly down-regulated in smokers (Figure 2G). The list of additional top predicted genes can be found in Supplementary File S1 (see Supplementary Materials).

### 2.2. Temporal Changes in Mutational Landscape of Long-Term Exposure to Waterpipe Smoke in Lung Cancer Cell Lines Genomes

While high-throughput sequencing studies have previously reported whole-genome analysis at the genomic, transcriptomic and proteomic levels in samples from smokers compared to non-smokers [25,26,27,28,29,30], as well as in samples from lung cancer [31,32], to date, the genomic landscape in long-term WPS-exposed lung cancer cell lines remains unknown.

**Figure 3:**
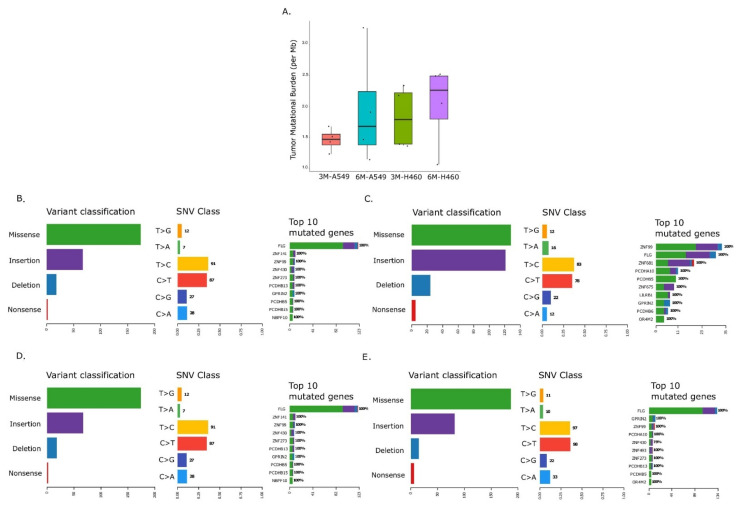
Mutational analysis of long-term WPSC treatment of lung cancer cell lines. WPSC treatment led to an increase in the tumor mutational burden (TMB), represented as an increase in total mutations per megabase in both A549 and H460 cell lines (**A**). Summary of mutations in A549 and H460 cell lines, respectively, treated with WPSC for 3 months (**B**,**D**) and 6 months (**C**,**E**).

We used NGS-based whole genome sequencing to analyze mutational burden in A549 and H460 cell lines exposed to 0.5% WPSC for up to 6 months. Our results indicate an overall increase in TMB (per Mb) in 3-month-treated samples that increased further in 6-month-treated samples (1 < medianTMB < 4; *p*-value < 0.05, Wilcoxon Rank Sum test) (Figure 3A). We observed that there were more missense mutations and frameshift insertions, compared to frameshift deletions and nonsense mutations in both cell lines. An overall increase in the frame shift insertions in the 6-month-treated samples was observed compared to 3-month-treated samples; these were limited to 1 to 4 bps insertions of C or T of homopolymer lengths. No insertions of >1bp as repeats were found for either of the cell lines (Figure S1, A549 and Figure S2 H460). When we analyzed missense mutations, we observed a greater number of transitions compared to transversions, specifically C -> T and T -> C mutations (Figure 3B–E); these are not enriched at APOBEC target sites (the TCW motif). Finally, we analyzed the distribution of single nucleotide variants (SNV) across different chromosomes as a function of log_10_(inter SNV event distance). This allowed us to look for patterns of localized hypermutations or Kataegis, known to be implicated in various cancer types. We observed an increase in Kataegis on chromosome 19 in 6-month-treated A549 and chromosome 1 in 6-month-treated H460 when compared to the respective three month treated samples (Figure S3A–D). Together, these data indicate that WPSC exposure over time leads to an increase in tumor mutational burden.

Mutations in cancer genes have been shown to occur at certain hot spots, providing an adaptive advantage to the cells and thereby getting positively selected during clonal evolution. We analyzed the genes that are mutated in response to WPS treatment in both cell lines. We investigated gene mutations with a large spatial clustering using clusterScore at z-score >2 and FDR < 0.01 (see Section 4). A clusterScore of 1 indicates the presence of reported mutations within clusters across all samples. In A549, ZNF99, PCDHB5, GPRIN2 and LILRB1 had clusterScores > 0.7 (cluster numbers: ≥5, 2, 2 and ≥1). In H460, FLG, PCDHA10, GPRIN2 and PCDHB13 had clusterScores > 0.7 (cluster numbers: ≥25, ≥1, >2 and ≥1). A complete breakdown of the clustering can be found in the Tables S1–S4.

**Figure 4:**
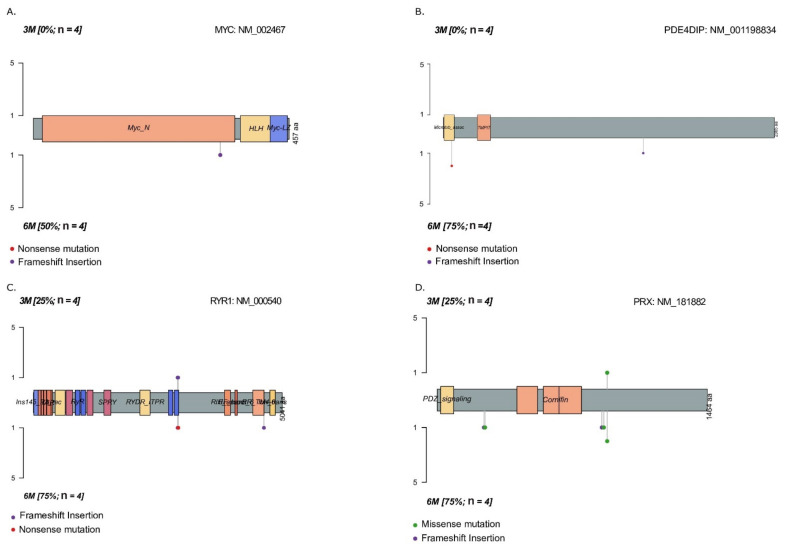
Mutational landscape of long-term WPSC treatment of lung cancer cell lines. Genes differentially mutated in 6-month-treated H460 samples were MYC (mutation rate 50%) and PDE4DIP (mutation rate 75%) (**A**,**B**), and genes differentially mutated in 6-month-treated A549 samples were RYR1 and PRX (75% mutation rate each) (**C**,**D**).

Next, we performed pathway analysis to identify differentially mutated oncogenic genes following long-term WPSC exposure. We identified genes in the MYC and NOTCH pathways that were mutated in 6-month-treated H460 samples but not in 3-month-treated samples (Figure 4); these were MYC (mutation rate 50%) and PDE4DIP (mutation rate 75%), due to frameshift insertions and nonsense mutations. Mutations in these genes have not been reported previously as per the variant effect predictor (VEP) database. Genes that were differentially mutated in 6-month-treated A549 samples were PRX and RYR1 (75% mutation rate each) due to missense and nonsense mutations, and frameshift insertions. One missense mutation observed in PRX gene-rs268673: Ile921Met had already been reported in the dbSNP database, with a known moderate impact; however, all the additional mutations we observed in PRX and RYR1 genes have not been reported previously to the best of our knowledge. Additional differentially mutated genes can be found in Figure S4.

In sum, we found an increase in TMB in six-month, WPS-treated cancer cell lines, with an increase in C to T and T to C transitions and frameshift insertions of 1–4 bp homopolymer lengths. We identified genes with an adaptive potential, with GPRIN2 being common across both cell lines. Finally, we found differentially mutated genes in response to the long-term exposure of WPS, including genes from the MYC and NOTCH pathways.

### 2.3. Smoking Is a Key Determinant of TMB of Lung Adenocarcinoma Patients

Although cancer cell lines are widely used as an in vitro experimental model in cancer studies, they do not constitute an ideal model for primary tumors due to differences in the microenvironment [33]. Furthermore, studies using smoke extract on cell lines do not parallel human smoking parameters because of variabilities in concentration and in the cell-to-smoke exposure interface in vivo vs. in vitro. In line with this, studying primary lung tumors and their microenvironment in smokers and non-smokers at a molecular level assumes a level of importance. We thus investigated lung adenocarcinoma (LUAD) molecular signatures in affected patients with long-term active exposure to smoke and compared them to patients who had not had any active exposure to smoke in their life. Because there are no studies on patients solely consuming WPS, as which would have been most relevant to our study, we took advantage of the large-scale TCGA molecular dataset on LUADs to compare the differences in molecular signatures in lifelong non-smokers versus tobacco smokers.

We divided the patients into two groups based on their smoking status: (1) life-long non-smokers and (2) smokers. We first compared the TMB in smokers and non-smokers affected with LUAD. A higher TMB was observed in smokers compared to non-smokers

medianTMB_smokers_ = 4.5,medianTMB_non-smokers_ = 1.09

**Figure 5:**
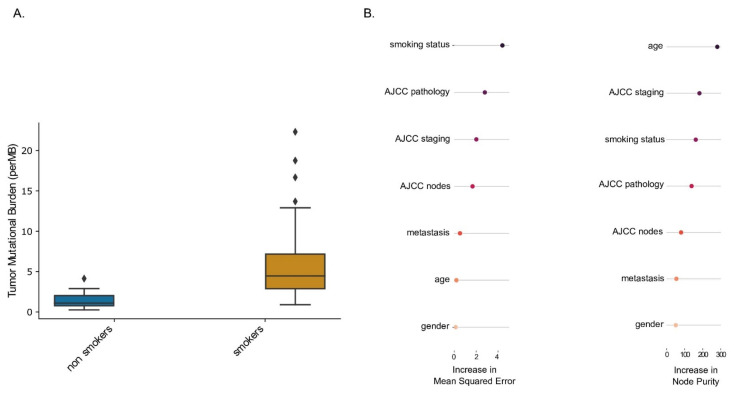
Tumor mutational burden increases in smokers affected with lung adenocarcinoma. (**A**) Analysis of the TCGA molecular dataset on lung adenocarcinoma patients was performed comparing molecular signatures in lifelong non-smokers versus tobacco smokers. A higher TMB was observed in smokers compared to non-smokers (medianTMB_smokers_ = 4.5, medianTMB_non-smokers_ = 1.09; *p*-value = 4.13 × 10^−10^ Wilcoxon Rank Sum test with continuity correction). (**B**) Random forest model-based feature importance technique was performed to assess the effect of smoking status alone while controlling for the listed confounding factors.

*p*-value = 4.13 × 10^−10^ Wilcoxon Rank Sum test with continuity correction) (Figure 5A). In addition to the smoking status, several factors such as age, gender, tumor stage and metastasis status could affect the overall TMB state. We used two random-forest-model-based feature importance techniques, Increase in Mean Square Error (IncMSE) and Increase in Node Purity (IncNodePurity), to assess the effect of smoking status alone while controlling for these confounding factors. We observed that smoking status remained among the top three important features that are important for TMB prediction with IncMSE = 4.5 and IncNodePurity = 161 (Figure 5B).

### 2.4. Smoke Exposure Is Associated with a Reprogramed Tumor Immune Microenvironment

The immune microenvironment could have a key role in determining immunotherapy outcomes. To better understand these microenvironmental factors, we focused on four major signatures: (1) immune cell fractions associated with immunotherapy response, (2) the success of T-cell infiltration into tumors, (3) T-cell dysfunction within the tumor microenvironment and (4) the expression of immune checkpoint genes.

**Figure 6:**
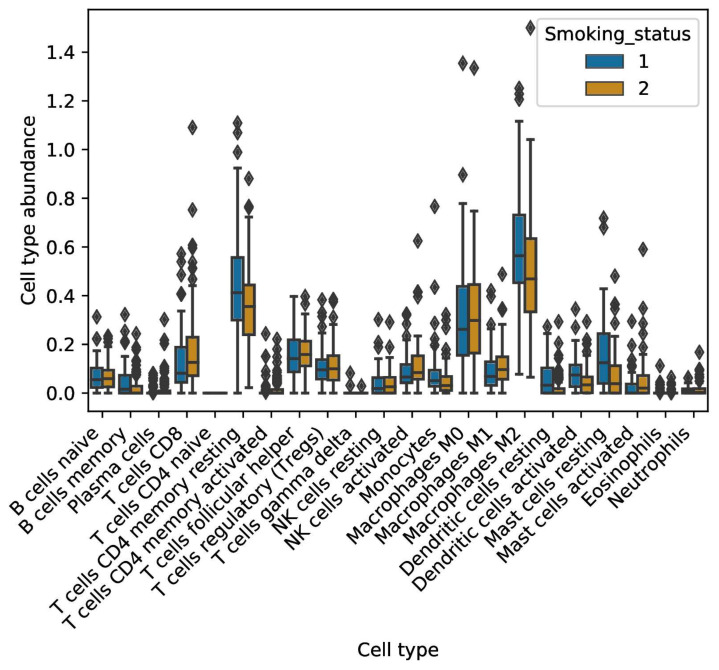
Immune profile of smokers affected with lung adenocarcinoma. Various immune cells profiles were analyzed in non-smokers (1) compared to smokers (2).

The digital cytometer CIBERSORTx was first applied to examine immune cell fractions residing in smokers vs. non-smokers (Figure 6). When compared to smokers, non-smokers had a higher fraction of the antigen-presenting dendritic cells (Wilcoxon rank sum test, *p*-value = 9.369 × 10^−5^). However, they also had a higher fraction of the immunosuppressive M2-polarized macrophages (Wilcoxon rank sum test, *p*-value = 0.0048). Regarding smokers, they displayed higher cell fractions of anti-tumor M1 macrophages (Wilcoxon rank sum test, *p*-value = 0.05), as well as NK cells (Wilcoxon rank sum test, *p*-value = 0.017). Finally, we observed a higher cell fraction of Cytotoxic T lymphocytes in smokers when compared to non-smokers (Wilcoxon rank sum test, *p*-value = 0.04). No differences were found in other B-cell and T-cell fractions, including T-regulatory cells, with the latter being associated with immunosuppressive effects.

**Figure 7:**
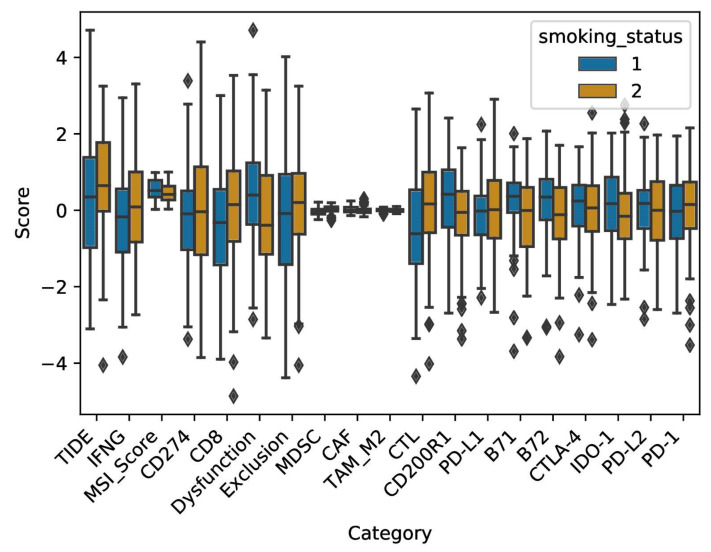
Functional state of infiltrating CTLs and Immune checkpoint genes expression. The functional state of infiltrating CTLs and their degree of exclusion from the tumor microenvironment were evaluated using TIDE. Furthermore, expression of eight immune checkpoint genes was analyzed in LADC patients with a history of smoking. (1) non-smokers; (2) smokers.

To evaluate the functional state of infiltrating CTLs and their degree of exclusion from the tumor microenvironment, the TIDE (Tumor Immune Dysfunction and Exclusion) algorithm, TIDEPY, was utilized (Figure 7). First, we observed a higher score of Cytotoxic T lymphocytes in smokers when compared to non-smokers (Wilcoxon rank sum test, *p*-value = 0.0036). This was calculated using five genes, CD8A, CD8B, granzyme A, granzyme B and Perforin expression. This effect remains after controlling for confounding factors such as age and gender using a multiple linear regression (MLR) model fit (coefficient_smoking status_ = 0.67, 95% confidence interval = (0.22, 1.12), *p*-value = 0.003). Of interest, a lower read out for T-cell dysfunction score was observed in smokers as compared to non-smokers (Wilcoxon rank sum test, *p*-value: 0.0096). Regarding T-cell exclusion, which was based on the presence of immune-inhibitory cells (Cancer Associated Fibroblasts (CAFs), myeloid-derived suppressor cells (MDSCs) and M2 macrophages), no differences could be observed between smokers and non-smokers (Wilcoxon rank sum test, *p*-value = 0.1). Other markers such as microsatellite instability (MSI) and interferon gamma (IFN-γ) were also analyzed for differential expression between the two groups. There was no difference in IFN-γ levels between smokers and non-smokers (Wilcoxon rank sum test, *p*-value = 0.3). Furthermore, a higher median score of MSI, a result of defective mismatch DNA repair, was observed in non-smokers than smokers (Wilcoxon rank sum test, *p*-value = 0.028), albeit the distributions were broad.

Finally, the expression of immune checkpoint genes (ICGs) in both groups was analyzed (Figure 7). Expression was measured in terms of z-score (see Section 4. for details). While there was no difference in the expression levels of PD-1 (Wilcoxon rank sum test, *p*-value = 0.24), PD-L1 (Wilcoxon rank sum test, *p*-value = 0.32), PD-L2 (Wilcoxon rank sum test, *p*-value = 0.66) and CTLA-4 (Wilcoxon rank sum test, *p*-value = 0.52) between smokers and non-smokers, higher expression levels of co-inhibitory molecules B7-1 (Wilcoxon rank sum test, *p*-value = 0.0025) and B7-2 (Wilcoxon rank sum test, *p*-value = 0.0174) were observed in non-smokers. Similarly, other suppressors of antitumor responses had higher expression in non-smokers than smokers, namely, IDO1 (Indoleamine 2, 3-dioxygenase 1) (Wilcoxon rank sum test, *p*-value = 0.27) and CD200R1 (Wilcoxon rank sum test, *p*-value = 0.001). Our analyses of TCGA data provide support for smoking in modulating lung adenocarcinoma patient’s tumor microenvironment resulting in immune cell landscape variations. These would constitute potential key targets in therapy modalities.

## 3. Discussion

Accumulated evidence indicates that smoke plays a central role in the evolution of tumor ecosystem and immune escape mechanisms by tumor cells through its impact on immune plasticity and tumor heterogeneity. In this regard, we had previously observed that treating lung cancer cell lines with WPSC resulted in an increase in DNA damage [11]. Here, we asked whether WPSC interferes with the autophagic process and how this may influence the immune landscape in the lung of smokers. Our current data indicate an increase in apoptosis at early WPSC exposure times, confirming other published works [14,34,35,36,37]. Furthermore, we noted an activation of autophagy following WPSC treatment. Autophagy inhibition resulted in an increase in apoptosis, highlighting a role for autophagy in sustaining cancer cell survival. The cells that escape apoptosis can either undergo autophagy or senescence. While elevated levels of autophagy induce cell death, inadequate autophagy can trigger cellular senescence [38], which we have previously shown is also induced following 8-day treatment with the same concentrations of WPSC [11]. While DNA damage potentiates different repair mechanisms to restore the damaged DNA, which, if unrepaired, would lead to the activation of cell death programs [39], autophagy has been shown to function in delaying apoptotic cell death in cancers as autophagy inhibition sensitizes cancer cells to chemotherapeutic drugs and/or ionizing radiation [32,40,41,42,43] and is also shown to play a role in the inhibition of the immune response in cancers with high TMB [44]. In WPSC-treated cells, we measured an increase in TMB in vitro; TMB has been observed in several cancers with DNA damage repair gene mutations [45,46,47]. While we did not analyze the DNA damage repair gene status in our study, we did observe an increase in TMB in cell lines exposed to WPSC from 3 to 6 months exposure. Our analysis of the TCGA LUAD dataset reaffirms our results, where we saw an increase in TMB in patients with an active smoking status. Other studies have also addressed the effects of tobacco smoking on normal as well as lung cancer and found this to be associated with an increase in TMB [48,49,50]. We analyzed the genes that were affected with mutations and divided them into two categories: (1) genes with specific mutational hotspots that arise because of the treatment across all samples and (2) differentially mutated genes that only get mutated as the mutational burden increases in the 6-month-treated samples. Genes such as zinc finger protein 99 (ZNF99), a gene found to be mutated in NSCLC with resistance to etoposide [51], and FLG, a highly mutated driver gene found in lung cancer [52], GPRIN2 and PCDHB13 that has been found to be downregulated in NCSLC and that negatively correlated with pathological grade [53], were mutated in all treated samples in both cell lines with a 100% mutation rate. In addition, discrepancies in the results obtained in our study with respect to WPS exposure to cell lines and patients’ data could be due to the significant role of the TME in modulating cancer cell behavior.

WPS exposure could be modulating several biological pathways that would act upstream of DNA damage. Exposure to WPS induces significant alterations in inflammatory cytokines and oxidative stress markers in mice [8,9,10,54,55]. WPS exposure also induces hypoxia [56]. Reactive oxygen species (ROS) could also be generated because of an increase in apoptotic cell death [57], which could generate a positive feed-back to further activate autophagy pathways [58].

Upon modeling-based analysis of TCGA lung adenocarcinoma RNA-seq datasets, we found an activation of autophagy in smokers. The most significantly affected genes were BNIP3, SESN2, TRIM22 and TNFSF10. BNIP3 expression results in the initiation of autophagy by disrupting the beclin1/Bcl-2 complex [59], and BNIP3 protein has been reported to be overexpressed in several cancer types and to participate in enhanced tumor growth [60]. SESN2/Sestrin 2 is a stress-inducible protein that is induced under hypoxic conditions and is reported to be associated with oxidative-stress-induced autophagy [61,62]; indeed, the occurrence of cancers is associated with significant downregulation of SESN2 [63]. Interestingly, TRIM22 stimulates autophagy by promoting BECLIN 1 expression [64] and has also been shown to play a role in driving tumor growth and progression [65]. TNFSF10/TRAIL could induce autophagy in certain cancer cells [66]. Our results suggest that the genes predicted by our model can correctly classify smokers as smokers but could also misclassify non-smokers as smokers. This low accuracy of 0.65 (C.I:(0.5,0.78)) is due to excluding non-autophagy related genes in our analysis. Nevertheless, future treatment interventions based on the autophagy genes could be designed for smokers with a higher confidence than for non-smokers. The limitation of our analysis is that this dataset was analyzed for gene expression in smokers of any devices (cigarettes and others), due to the non-availability of studies that include patients consuming WPS alone.

Several studies have shown evidence for the significant role for autophagy in the response to therapeutic treatments in cancers [67]. Because autophagy induction could be associated with resistance to therapy, concomitant targeting of autophagy pathways synergizes with cancer therapeutic drugs to enhance cell death [67,68,69]. On the other hand, pro-autophagic drugs have been used successfully to enhance apoptosis in resistant cells [67]. This is due to the turning on of autophagic cell death mechanisms. Our in vitro data support the mechanism that autophagy is important to maintain cell survival, however the plastic nature of tumor cells and their continuous plasticity in response to their microenvironment may require regular monitoring to assess more effective treatment strategies. How WPS alone affects the autophagy response and the genetic landscape in lung adenocarcinoma patients compared to non-WP smoker patients has yet to be fully elucidated.

Unraveling the changes in the immune microenvironment in lung adenocarcinoma patients with a history of smoking could enhance our understanding of factors that could contribute to predicting response to immune checkpoint inhibitors. While various biomarkers of response have been validated and are being used in the clinic, the absence of efficacy in a fraction of patients underlines the need for further studies. We thus investigated the immunological landscape in LUAD patients with a history of smoking. We found a higher TMB, NK-cell infiltration, CD8+ T-cell fraction and lower dysfunction level in smokers as compared to non-smokers, even after controlling for various confounding factors. On the other hand, non-smokers seemed to display a more immunosuppressed state, with a higher infiltration of M2 pro-tumor macrophages. Our findings are in agreement with a recent study that showed that NSCLC patients who are previous or current smokers had a higher TMB and neoantigen load, accompanied by a higher infiltration of immune cells, compared to those classified as never-smokers [70]. However, unlike previous studies similar to ours, using statistical models like Random Forest and Multiple Linear Regression, we report for the first time that these results are not affected by, and are not a sole artifact of, other confounding factors; at least for the dataset that we analyze in the present study. Moreover, in accordance with our results, they also reported following mass cytometry (CyTOF) analysis of fresh NSCLC tissues, that smokers have a more immune-activated TME, while the TME of non-smokers is in an immunosuppressed or resting state [70]. Our findings further suggest a more complex relationship between smoking status and immune infiltration. A higher fraction of the immunosuppressive MDSC was present in smokers compared to non-smokers who displayed a higher infiltration of DCs and a higher level of MSI, which is a positive predictor of response to ICI. Considering other markers of response, no differences could be detected in expression levels of PD-L1, among other immune checkpoint genes. Autophagy activation has been shown to decrease the expression of histone deacetylases that downregulate PD-L1 expression [48], validating our findings. Interestingly however, B7-1, B7-2, IDO1 and CD200R1 had higher expression levels in non-smokers relative to smokers. Immune checkpoint inhibitors against IDO1, which negatively impacts T-cell differentiation, are currently being investigated in clinical trials [20]. Our results would suggest better efficacy of such agents in non-smokers compared to smokers. It is important to note that our findings are all based on in silico analysis of a single dataset and would require further validation in independent cohorts of lung adenocarcinoma. Nonetheless, they help shed light on the complexity that is the tumor immune microenvironment in smokers vs nonsmokers with LUAD and supplement the perspective that smoking is only a putative biomarker of response to immunotherapy.

Our results provide the first comprehensive analysis, to the best of our knowledge, that would help plan better treatment interventions targeted at LUAD patients with a history of smoking. We also call out the need for carrying similar TCGA studies including information specifically on patients exposed to WPS alone. Studying tumor microenvironment in patients with a history of smoking with a focus on autophagy could provide a stepping stone for novel directed immunotherapy approaches.

## 4. Materials and Methods

### 4.1. Waterpipe Smoke Sampling and Analysis

Waterpipe smoke sampling and analysis was described previously [11].

### 4.2. Cell Culture

A549 (gift from Prof Fathia Mami Chouaib, Gustave Roussy, Villejuif Cedex, France) and H460 cells (AddexBio C0016003 RRID:CVCL_0023, San Diego, CA, USA) were grown in complete RPMI 1640 Medium, (Gibco 61870010, Life Technologies, Warrington, UK) supplemented with 10% Heat Inactivated Fetal Bovine Serum (Gibco 10270-106 Life Technologies, UK), 1% Penicillin-Streptomycin (Gibco 15140-122, Life Technologies, UK) and 1% sodium pyruvate (Gibco 11360-039, Life Technologies, UK). We tested and confirmed that all the cell lines were mycoplasma-free.

### 4.3. MTT Assay

MTT was obtained from Abcam (MTT Assay Kit, Abcam ab211091, Cambridge, UK). A549 and H460 cells were seeded at a density of 0.5–1 × 10^4^ cells/mL in 96 well plates. The plates were then treated with concentrations of 0.2% WPSC for 24, 48 and 72 h. 200 µM hydrogen peroxide treatment was used as a positive control for 25 min. Cells were treated with 100 nM Bafilomycin A1 (Cell Signaling, 54645, Danvers, MA, USA), where indicated for 1 h prior to WPSC treatment. 20 μL of MTT solution (5 mg/mL) was added to each well and the cells were cultured for another 2 h. The treatment medium was then discarded and 50 µL of serum-free media and 50 µL of MTT Reagent were added together into each well and the plate was incubated at 37 °C for 3 h. Following incubation, 150 µL of MTT solvent was added to each well. The plate was covered in foil and agitated on an orbital shaker for 15 min then read at 590 nm using a microplate reader (BioTek Epoch 2, Winooski, VT, USA). Cell proliferation rates were calculated by comparing with the control cells.

### 4.4. Flow Cytometry

Apoptosis assays were performed using APC Annexin V Apoptosis Detection Kit with Propidium Iodide (PI) (Biolegend, 640914 San Diego, CA, USA). Briefly, cells were plated at a density of 100,000 cells per dish in 35 mm dishes (Eppendorf 0030 700.112, Hamburg, Germany). Following WPSC treatment, the cells were collected at the indicated timepoints by trypsinization and subsequently washed with 1× PBS prior to labeling with Annexin V-APC and PI following the manufacturer’s protocol. Acquisitions of 20,000 cells were performed using a Biorad S3E Cell Sorter and data processed using the FCS Express flow cytometry program (De Novo Software, Pasadena, CA, USA). Annexin V-positive cells were classified as apoptotic.

### 4.5. Statistical Analysis

Statistical analyses were carried out using GraphPad Prism Software version 9.3.1 (GraphPad Software, Inc, San Diego, CA, USA). All data are expressed as means ± SEM. Significant differences were found using two-way analysis of variance (ANOVA) followed by correction for multiple comparison using Tukey test.

### 4.6. Antibodies Used in This Study

Mouse anti-human SQSTM1/p62 (D5L7G) (Cell Signaling, Danvers, 88588, MA, USA), rabbit anti-human GAPDH (Cell Signaling 2118, MA, USA) and rabbit anti-human LC3A/B (Cell Signaling 4108, MA, USA).

### 4.7. Immunoblotting

Cells grown in 6-well dishes were washed once with ice cold PBS 1× and lysed in 100 µL of RIPA (150 mM NaCl, 0.1% TX-100, 0.5% NaDOC, 0.1% SDS, and 50 mM Tris-HCl pH 8.0) with protease inhibitor cocktail (Sigma P2714, Burlington, MA, USA). Proteins were quantified following brief sonication, by Pierce BCA protein assay kit (Thermo Fisher 23225, Rockford, IL, USA), and 15–20 µg of proteins were loaded on 10% or 12% SDS-PAGE and transferred onto a nitrocellulose membrane (Sigma GE10600004, Burlington, MA, USA) at 80 Volts for 3 h. After blocking with 5% BSA in TBST (10 mM Tris, pH 8.0, 150 mM NaCl, and 0.5 % Tween 20) for 60 min, the membrane was washed once with TBST and incubated with the listed antibodies according to their data sheets.

### 4.8. Immunofluorescence

Cells were fixed in 4% paraformaldehyde (ThermoFisher Scientific 28906, Waltham, MA, USA) in 1× PBS for 10 min at room temperature. Cells were then washed with 1× PBS and permeabilized with 0.1% TX-100 in PBS for 15 min at room temperature. Prior to staining, cells were blocked in 2% BSA in 1× PBS for 1 h at RT. Cells were then stained with a primary and secondary antibody as per the data sheets followed by three 5 min washes after each antibody staining. Cells were then mounted on glass slides using Prolong gold antifade reagent (ThermoFisher Scientific P36930, MA, USA) and visualized on Zeiss LSM 800 with Airyscan.

### 4.9. Whole Exome Sequencing Variant Analysis

Whole exome sequencing (WES) was carried out for two non-small cell lung cancer cell lines A549 and H460. Each cell line was treated with water pipe smoke (WPS) and cultured for six months in two sets of biological replicates. Furthermore, two technical replicates were set up for a given biological replicate. Samples for sequencing for each set were collected at 3 months and 6 months. Untreated cancer cells were used as a control.

The QiaAmp DNA Mini Kit was used to extract genomic DNA (Qiagen, Hilden, Germany). Exome libraries were prepared from 100 ng of genomic DNA using Ion AmpliSeq™ Exome RDY kit (ThermoFisher Scientific, A38264, MA, USA). With 293′903 total amplicons, this kit covers almost 97 percent of the exonic regions. The samples were barcoded using Ion Xpress Barcode Adapter 1–16 kit (ThermoFisher Scientific, 4474009, MA, USA). The libraries were purified using CleanPCR (Clean NA, GC Biotech, Waddinxveen, The Netherlands). Library quantification was performed using the Ion Library TaqMan Quantitation Kit (ThermoFisher Scientific, 4468802, MA, USA). The libraries were loaded onto the chips using Ion Chef System (ThermoFisher Scientific, 4484177, MA, USA) by utilizing Ion 540 Chef Reagents. Two samples per chip were loaded in equimolar concentrations (40 picomolar) and were sequenced on Ion S5 XL sequencer (ThermoFisher Scientific, MA, USA). The raw data were aligned with the hg19 version of the genome using Ion Torrent Suite (TS) software, and the bam files were processed for variant calling using low-stringency somatic variant and indel calling.

VCF files were obtained as an output of the Torrent Variant Caller. The sample IDs in the vcf header column were changed to ensure uniformity in the downstream analysis. These files were indexed and merged using the respective commands bcftools index and bcftools merge from the package bcftools v1.10.2 [71]. The merging was done in order to create a treated condition and an untreated control pair file. The vcf2maf.pl perl script was used with --remap-chain, vcf-tumor-id, vcf-normal-id, --tumor-id and --normal-id options to obtain the Mutation Annotation Format or MAF files. The --remap-chain option allowed us to remap variants from hg19 to GRCh37 assembly. This was important to successfully run the variant effect predictor (VEP) v102.0 for annotating variants [72]. The id options helped distinguish which sample out of the pair obtained in the previous step was the control. The preprocessing pipeline till this point was automated in Python 3.7.9.

The maf files were further analyzed using the R package maftools v2.6.05 [73]. The analyses were carried out after normalizing the variants called in the treated cancer cells against the untreated cancer cells used as the control. This allowed us to focus on only those variants that emerged in the cancer cells post-stress treatment. Sigprofiler was used to report Indel types across all samples [74].

### 4.10. TCGA Analyses

TCGA Firehose Legacy bulk RNA-seq Expression profiles were downloaded from cBioportal for Lung Adenocarcinoma (LUAD) with ~500 patient samples per dataset. Patient populations were segregated based on their smoking status. Six ordinal categories represented the following meta-data: (1) lifelong non-smoker, (2) current smoker, (3) current reformed smoker for ≥15 years, (4) current reformed smoker for ≤15 years, (5) current reformed smoker (duration not specified) and (6) smoking history not documented. We carried out the entire analysis, which follows below, using two categories: (1) lifelong non-smokers and (2) current smokers.

### 4.11. Tumor Mutational Burden (TMB) Analysis

The maf format files were segregated into smokers and non-smokers. The tumor mutational burden was calculated using the maftools package in R. To assess the feature importance, we used two metrics: (1) “increase in Mean Squared Error” or IncMSE, and (2) “increase in Node Purity” or IncNodePurity. They were used since the model was trained using the Random Forest Regressor in the R package randomForest. Seven features were included for this analysis: smoking status, age, gender, metastasis state, AJCC staging, AJCC pathology and AJCC nodes.

### 4.12. Immune Cell Abundance Analysis

Tumor deconvolution or immune cell abundance analysis was carried out using CIBERSORTx [75]. LM22 was used as the signature matrix, and B-mode batch correction (bulk mode) was applied. Quantile normalization was disabled. The analyses were run for 100 permutations, each with an absolute mode. Immune cell fraction distributions were compared across patients with different smoking statuses. The non-parametric Wilcoxon rank sum test was used to check for statistical significance. Multiple hypothesis tests were carried out using the false discovery rate (FDR) < 0.05.

### 4.13. TIDEPY Analysis

The python package TIDEPY (https://github.com/jingxinfu/TIDEpy, accessed on 12 May 2021) [76] was used to calculate the tumor immune dysfunction and exclusion for two groups, smokers and non-smokers, with lung adenocarcinoma (same dataset as mentioned above). Normalization was carried out using log2(x + 1) transformation followed by average subtraction across all samples.

### 4.14. Immune Checkpoint Analysis

Eight immune checkpoint genes were included in the analysis: PD-1, PD-L1, PD-L2, B7-1, B7-2, CTLA-4, IDO-1 and CD200R1, based on the two recent studies [77,78] highlighting the most responsive ICGs in lung adenocarcinomas as compared to normal tissues. The log(TPM) values were extracted for patients with confirmed status of being either smokers or nonsmokers, and Z-scores were calculated for each gene:

Z-score = log(TPM)_GeneX, PatientX_ − Mean − log(TPM)_GeneX_

Standard-deviation − log(TPM)_GeneX_

Z-score ranges from −1 to +1. A negative value indicates downregulation, and a positive value indicates upregulation.

### 4.15. Autophagy Modeling Analysis

A list of 370 genes involved in autophagy was curated from Fang et al. [24]. To fish out the autophagy genes that were highly predictive of smoking status based on their differential expression, we used a random forest regression model approach. For this, we used GeneSrF (varSelRF) [79], a python-based utility, to predict the top autophagy genes. We also applied our own random forest model testing using the R package randomForest with two hyperparameter values, ntree = 1500 and mtry = 19, obtained such that the Out-of-bag error rate was minimized to 22%. We used three performance metrics for our model: accuracy, precision and recall. Feature importance was calculated using MeanDecreaseAccuracy and MeanDecreaseGini. Autophagy gene expression distributions were compared using the Z-score as described above.

## Data Availability

The code employed in carrying out the entire analysis, along with supporting files and information, can be found at https://github.com/Mohak91/Waterpipe_smoke_lung_cancer_study (accessed on 1 June 2022).

## Supplementary Materials

The following supporting information can be downloaded at: https://www.mdpi.com/article/10.3390/ijms23126848/s1.
