# Supplementary material for: Genomic Analysis of Waterpipe Smoke-Induced Lung Tumor Autophagy and Plasticity
Source: Int J Mol Sci. 2022 Jun 20;23(12):6848. doi: 10.3390/ijms23126848 (PMC9225041; doi:10.3390/ijms23126848)
Supplement: Supplementary file 1 [file ijms-23-06848-s001.zip › ijms-1741587-supplementary/Supplementary figure legends.pdf]

Supplementary figure legends

Figure S1. Indel mutational signature plots across all eight samples, 3 month treated and 6 month treated A549 cell line.

Figure S2. Indel mutational signature plots across all eight samples, 3 month treated and 6 month treated H460 cell line.

Figure S3. Rainfall plots showing the distribution of SNPs across 23 chromosomes in two cell lines A) 3 month treated A549, B) 6 month treated A549, C) 3 month treated H460 and D) 6 month treated H460. The vertical arrows represent regions of Kateagis or localized hypermutation. The y-axis shows the log_{10}(inter event distance).

Figure S4. Forest plots showing differentially mutated genes in 3 month treated versus 6 month treated samples in two cell lines A) A549 and B) H460.
