# Supplementary figures and images for: Genomic Analysis of Waterpipe Smoke-Induced Lung Tumor Autophagy and Plasticity
Source: Int J Mol Sci. 2022 Jun 20;23(12):6848. doi: 10.3390/ijms23126848 (PMC9225041; doi:10.3390/ijms23126848)

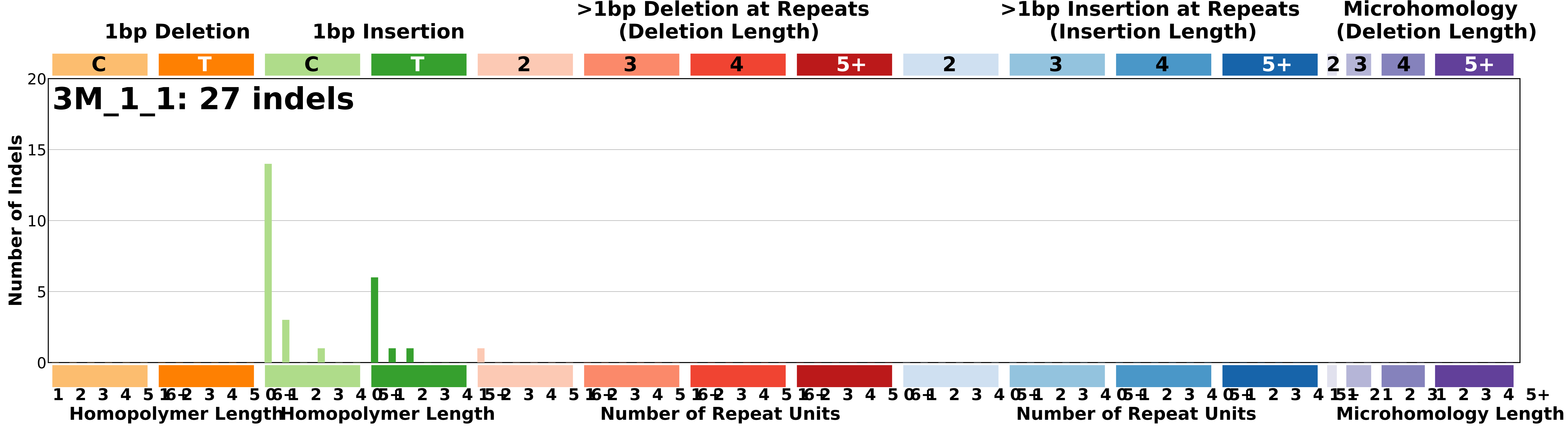

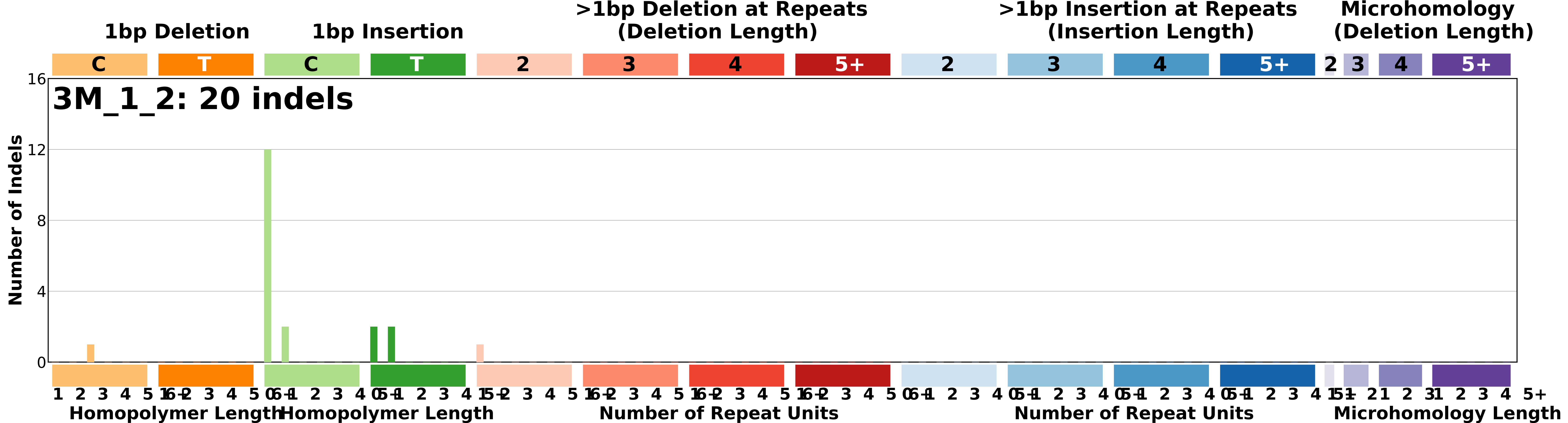

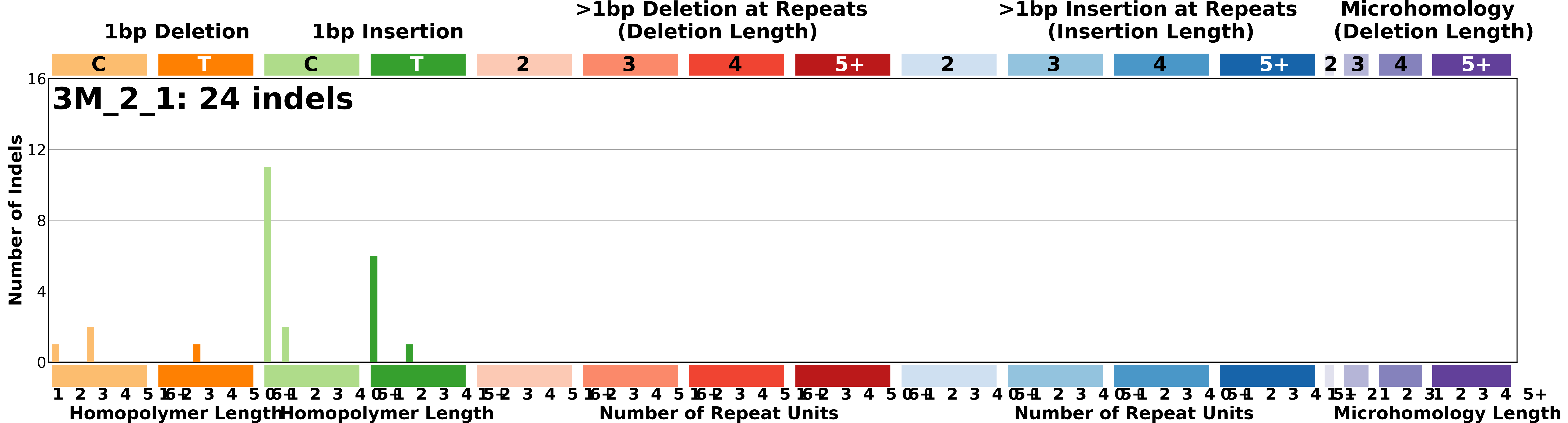

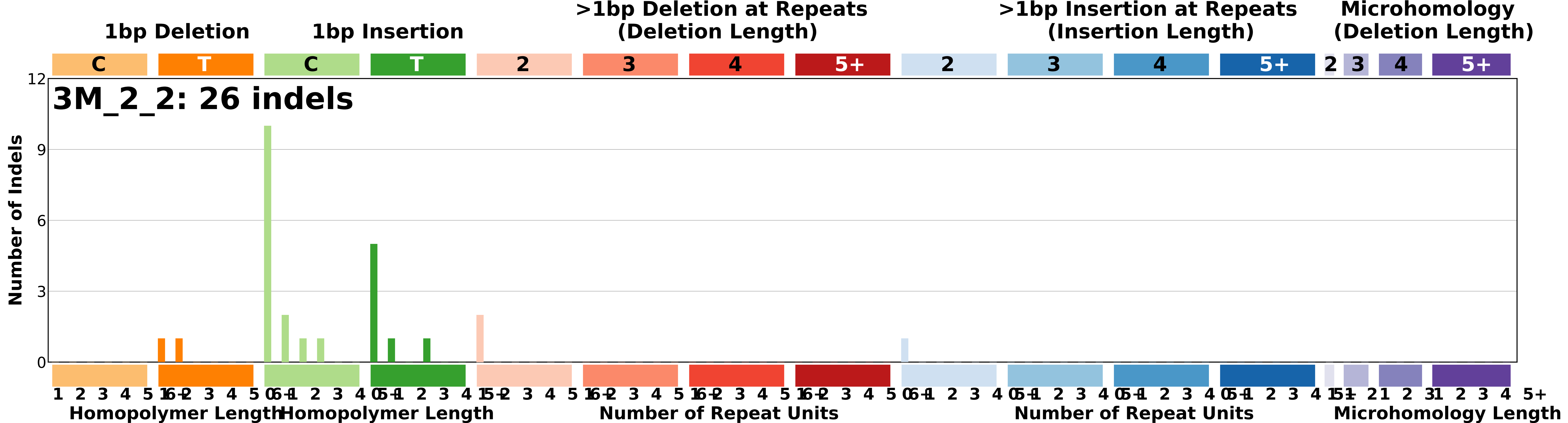

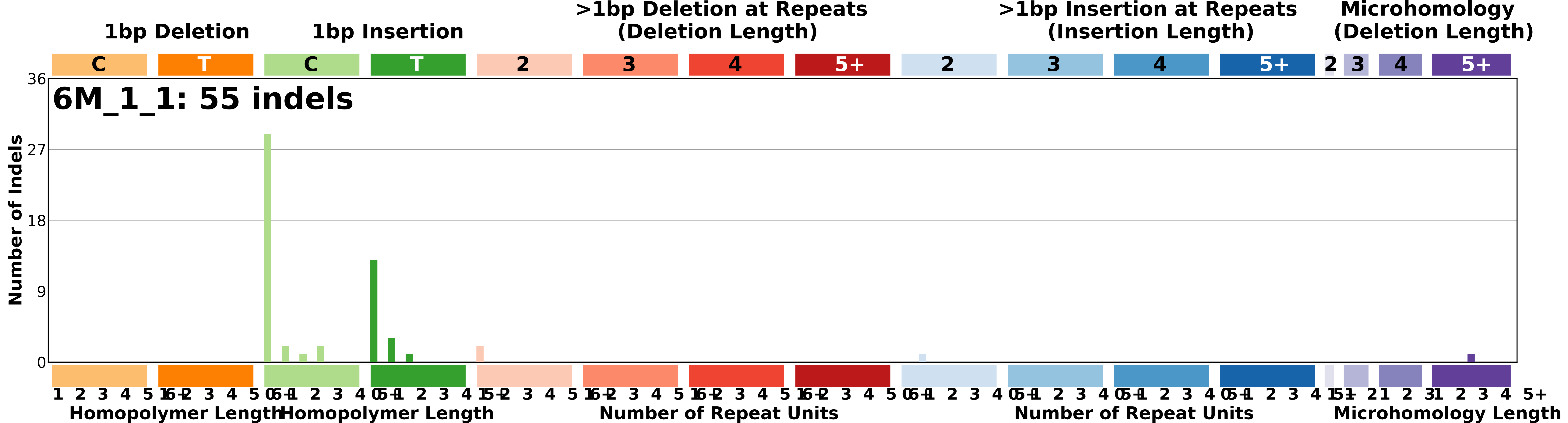

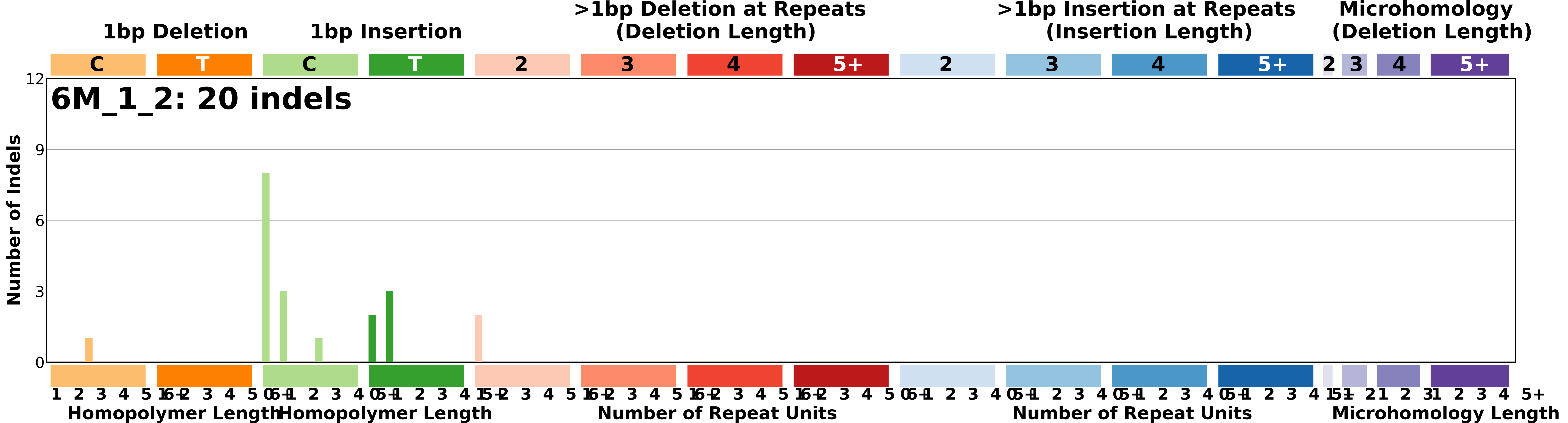

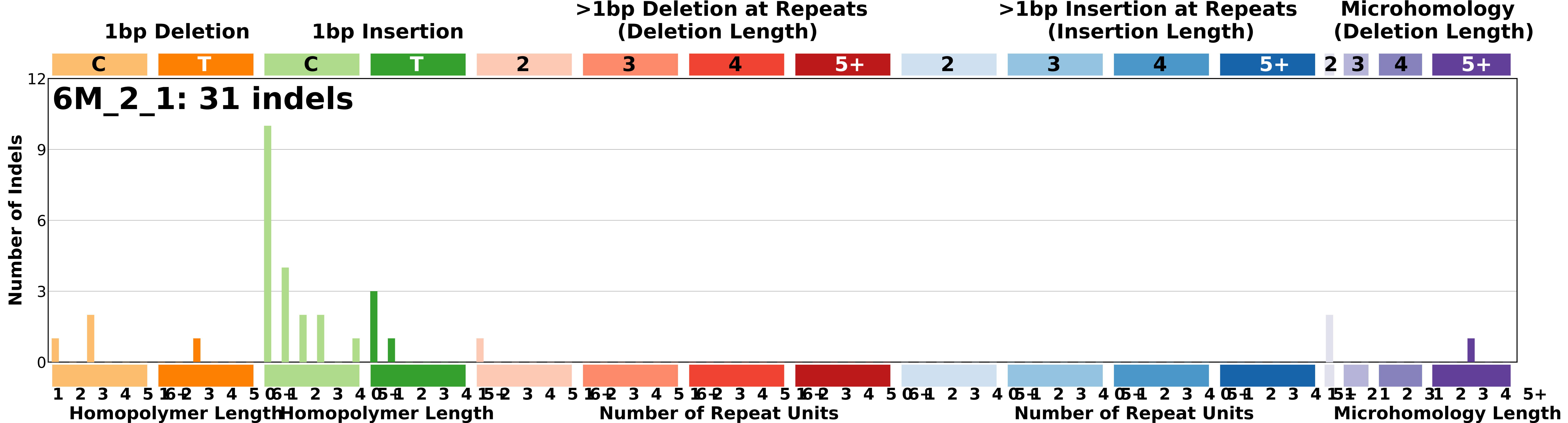

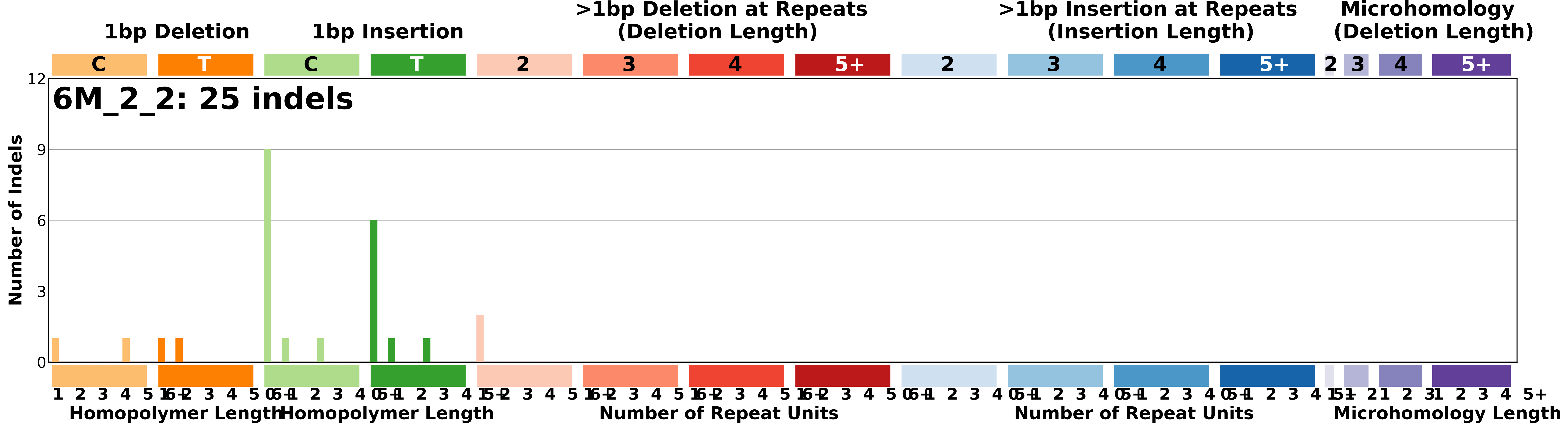

Supplement: Supplementary file 1 [file ijms-23-06848-s001.zip › ijms-1741587-supplementary/Supplementary_figures_files/Supp. Figures/FigureS1.pdf]

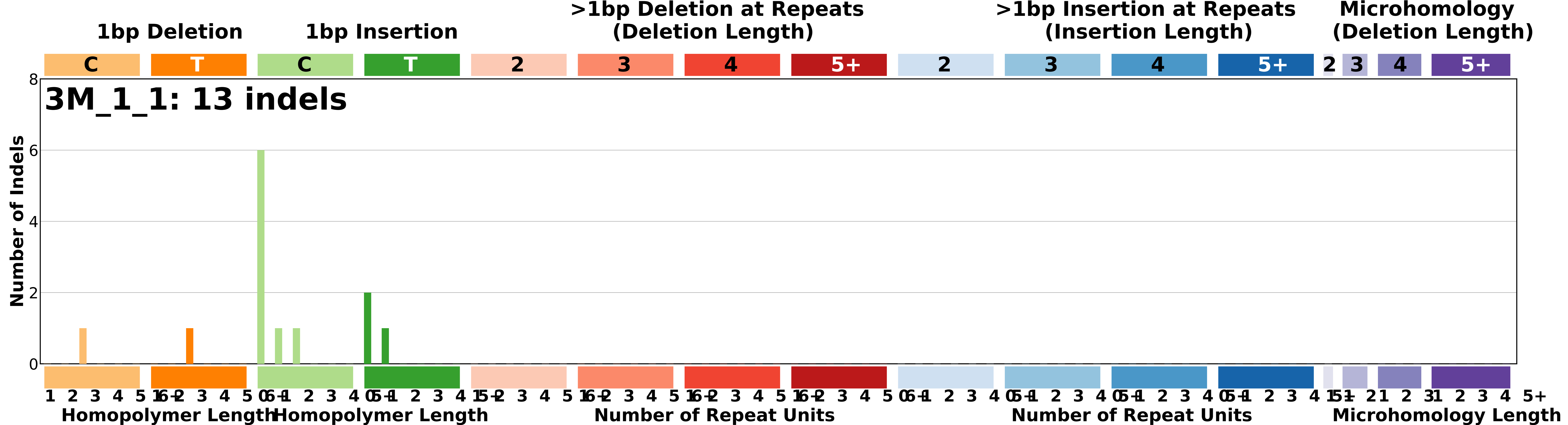

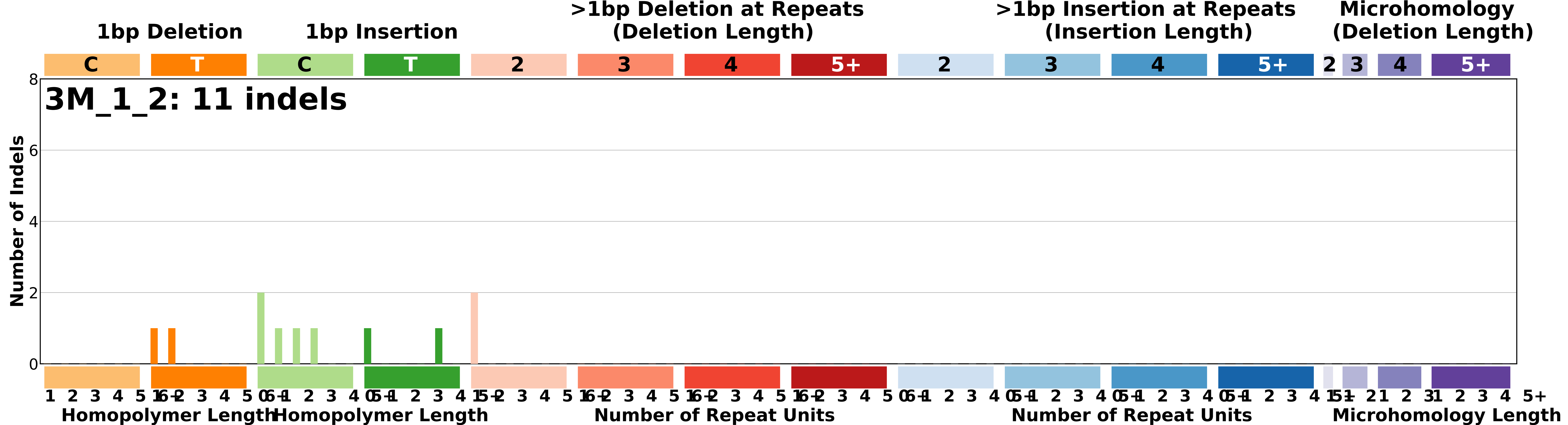

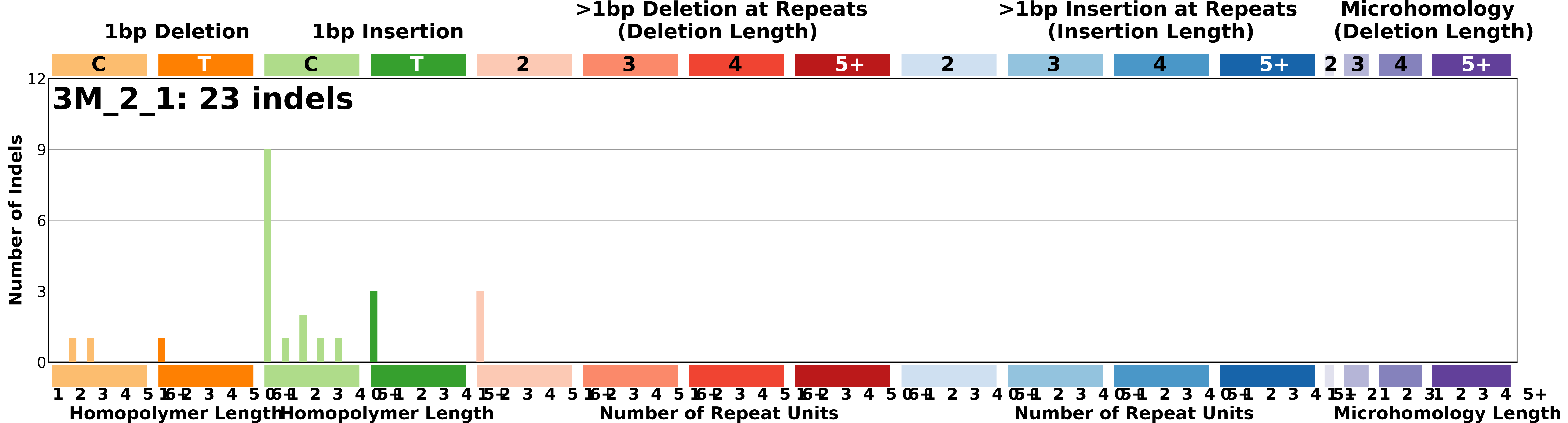

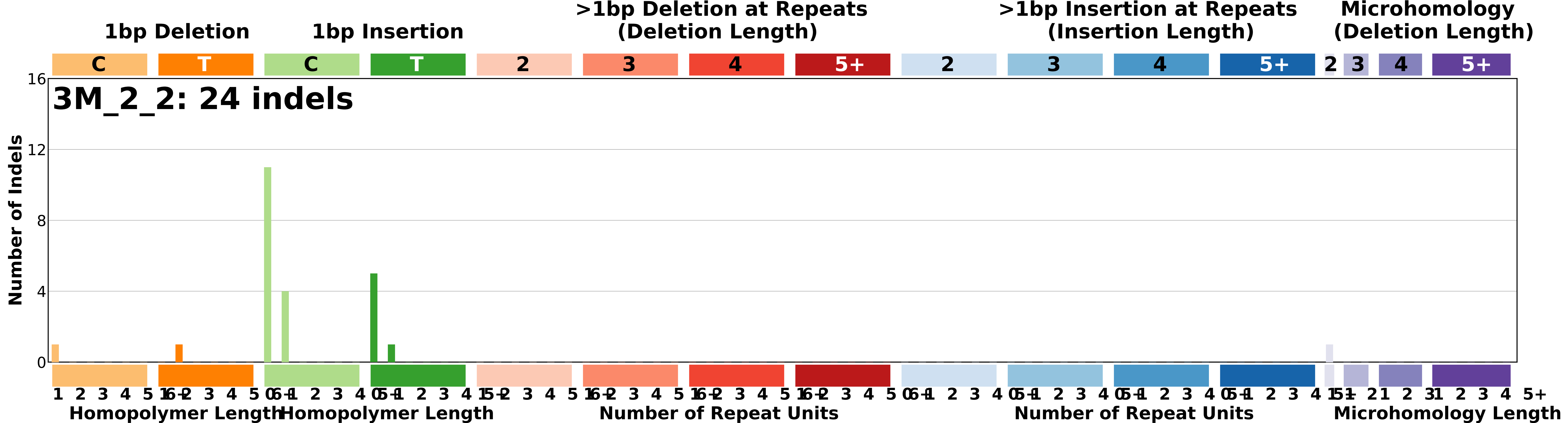

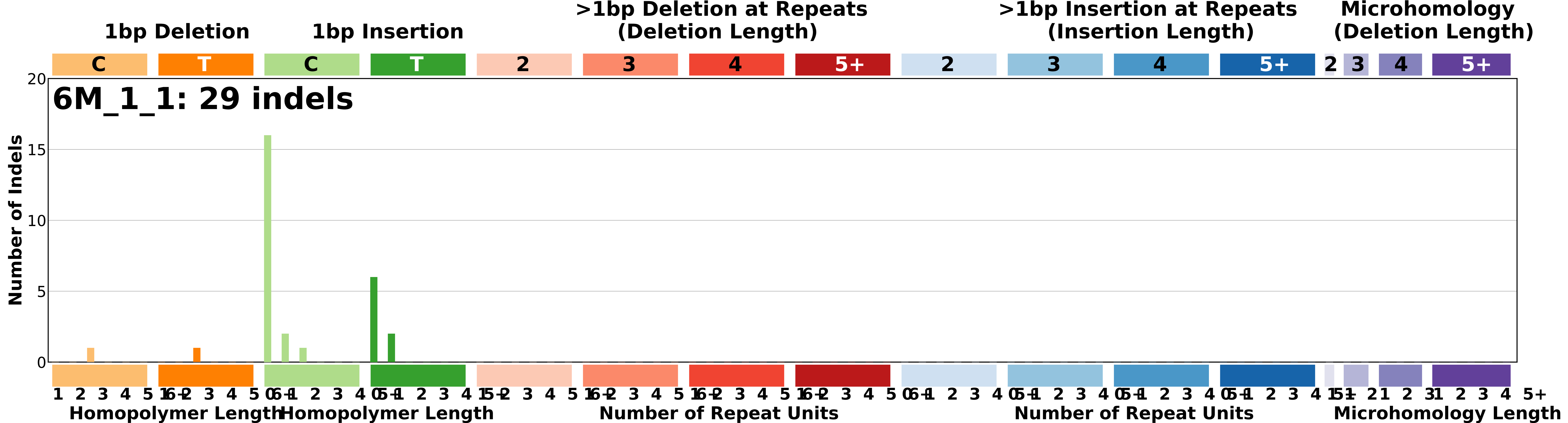

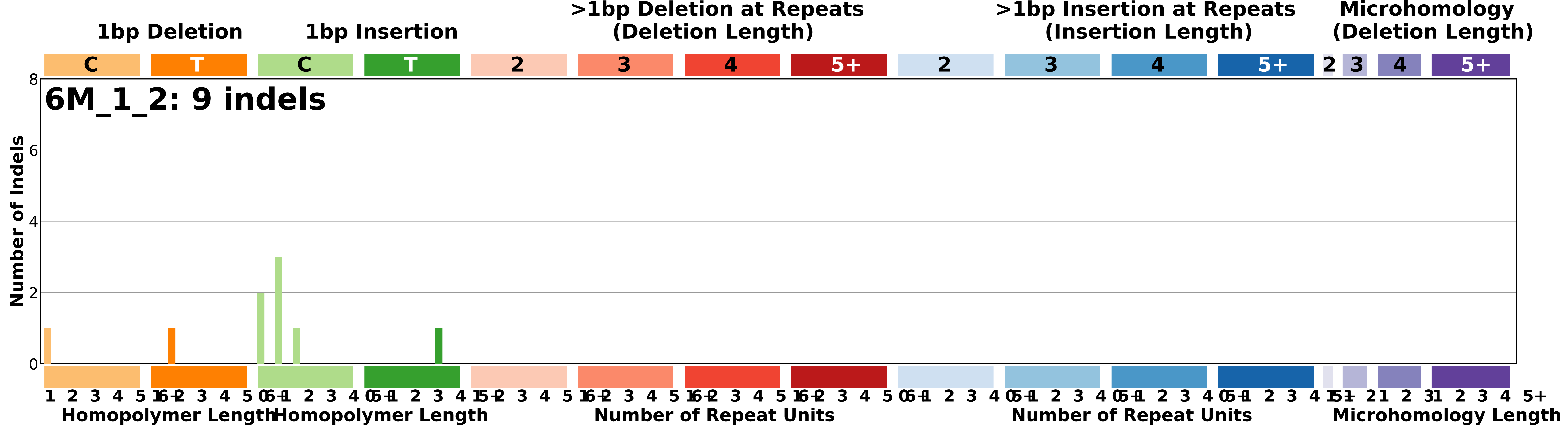

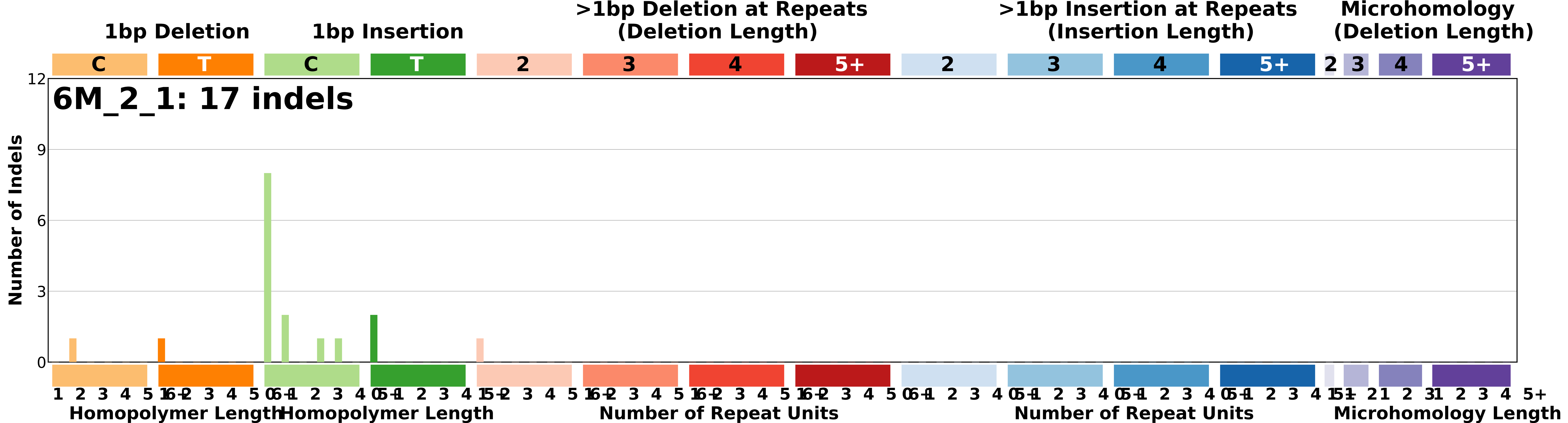

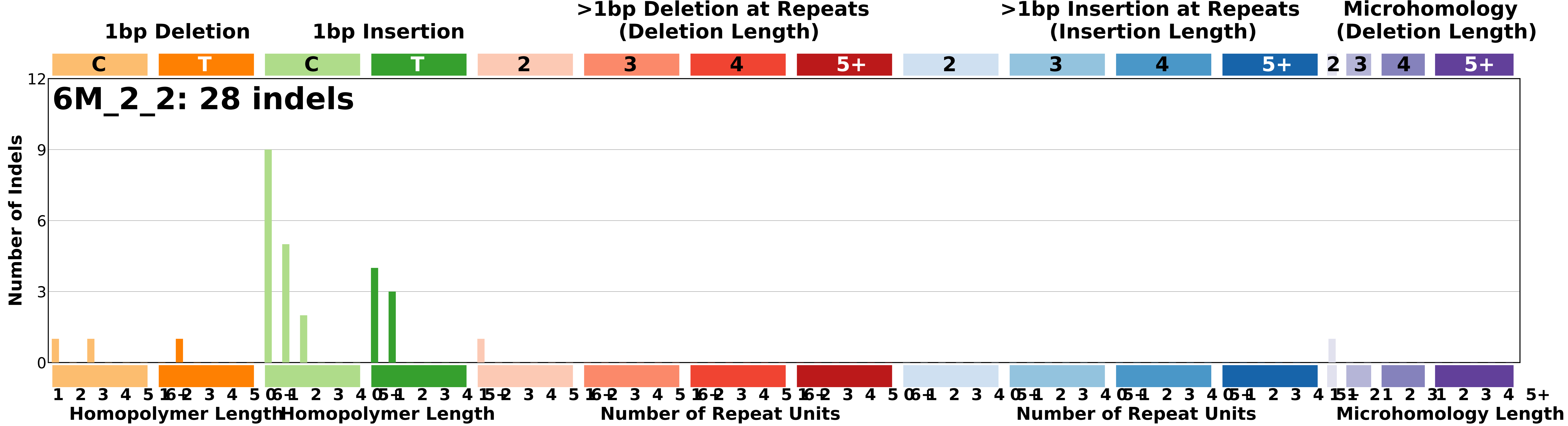

Supplement: Supplementary file 1 [file ijms-23-06848-s001.zip › ijms-1741587-supplementary/Supplementary_figures_files/Supp. Figures/FigureS2.pdf]

A)

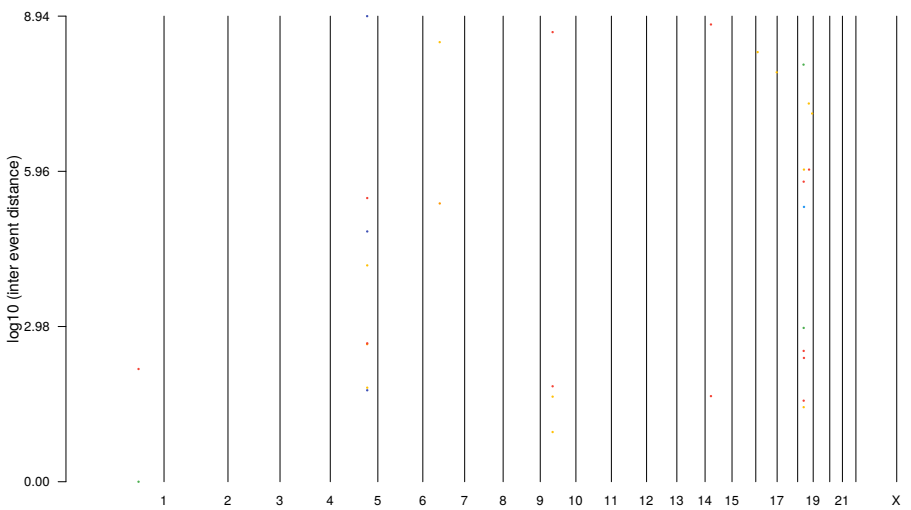

A549 - 3 month

B)

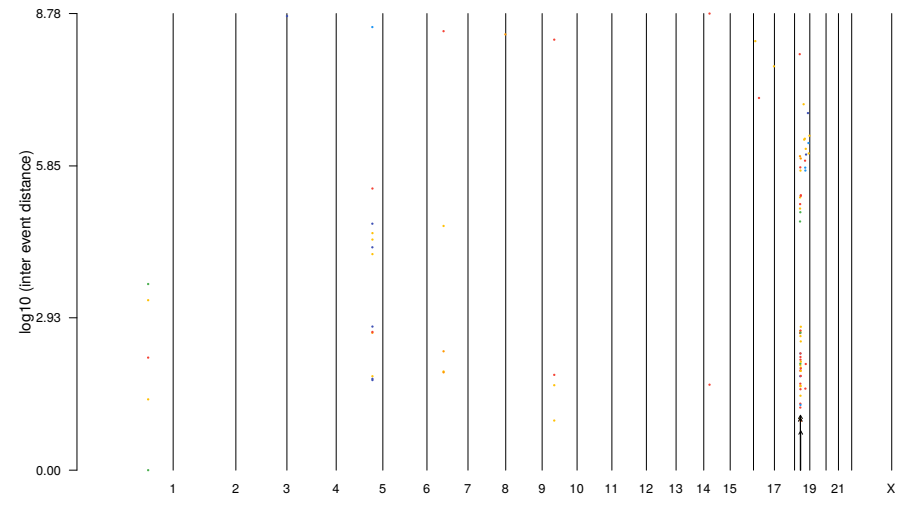

A549 - 6 month

C)

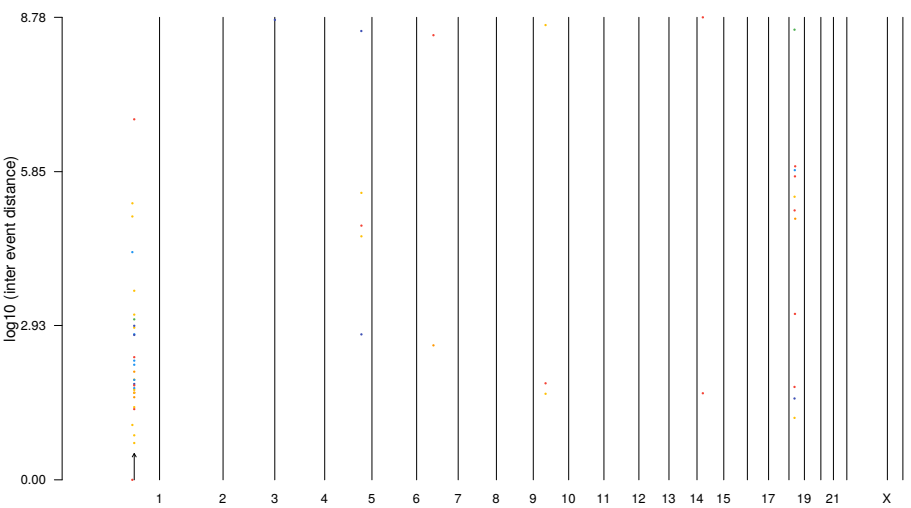

H460 - 3 month

D)

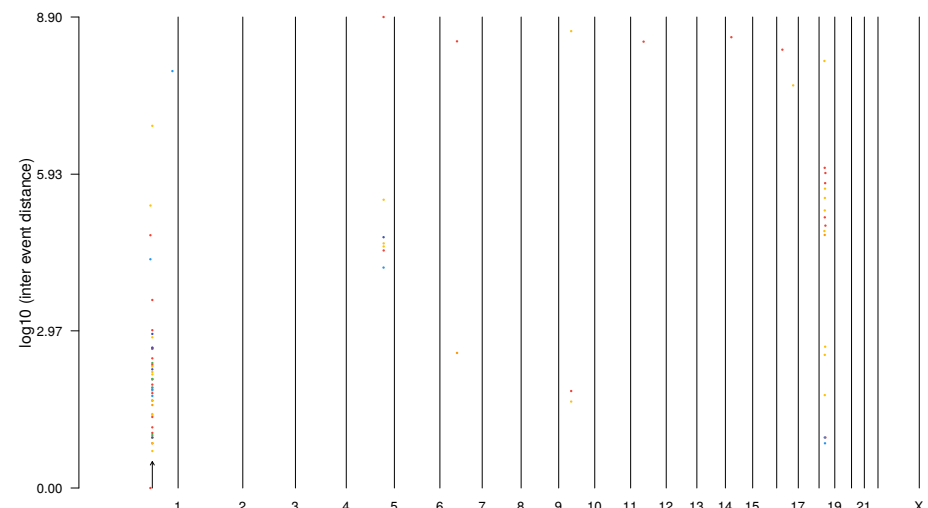

H460 - 6 month

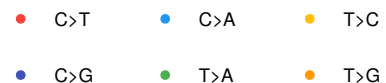

Supplement: Supplementary file 1 [file ijms-23-06848-s001.zip › ijms-1741587-supplementary/Supplementary_figures_files/Supp. Figures/FigureS3.pdf]

A)

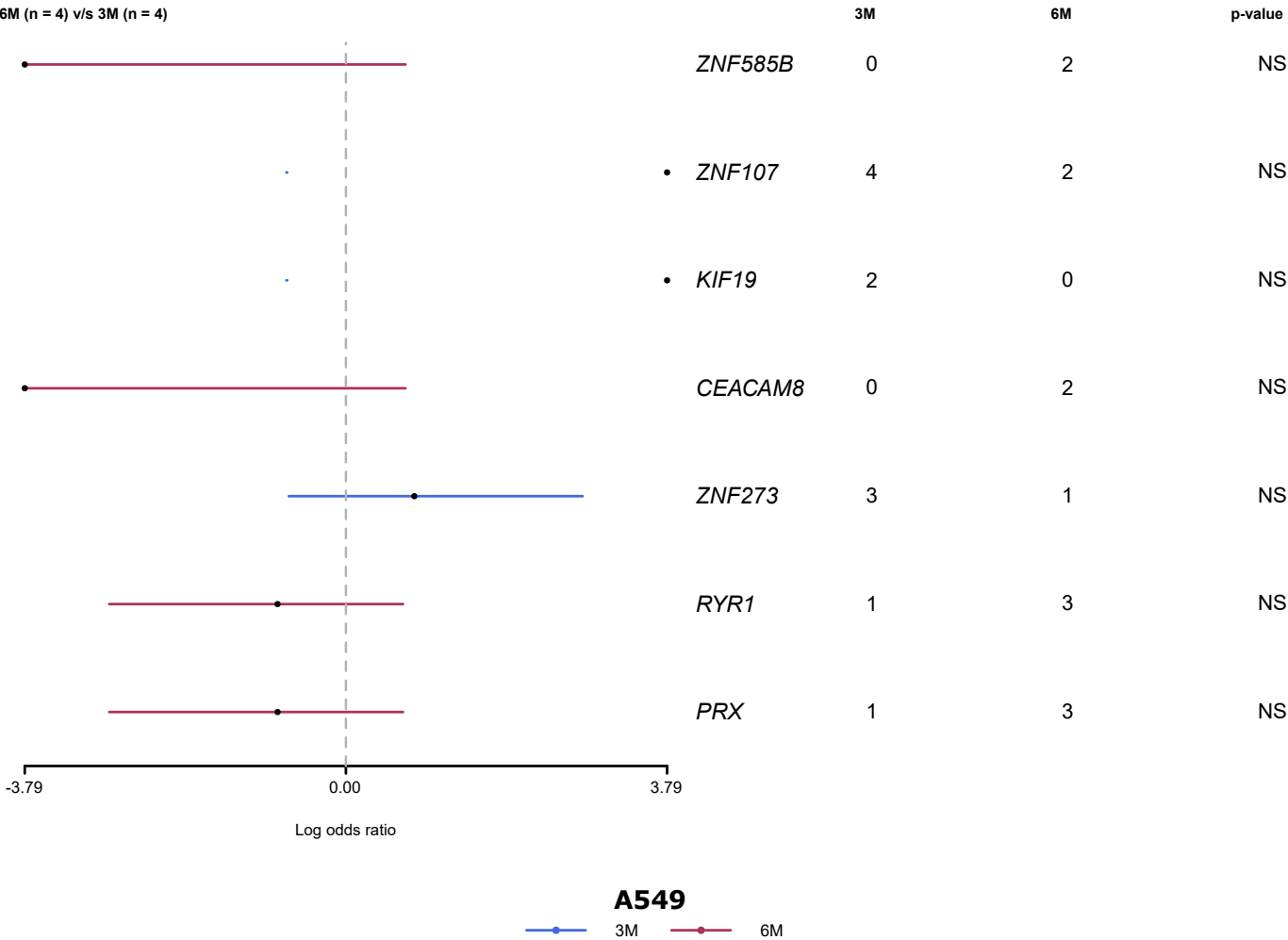

B)

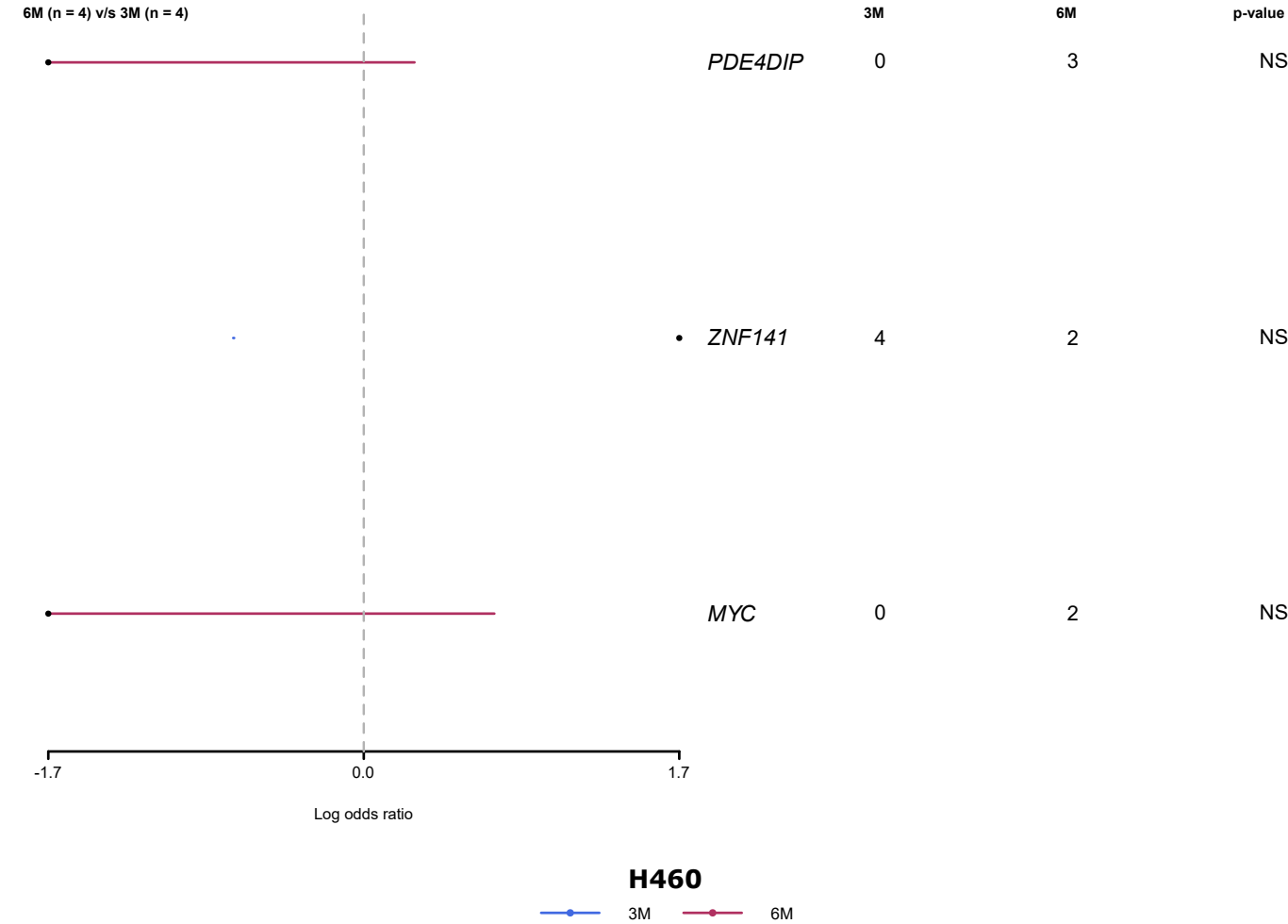

Supplement: Supplementary file 1 [file ijms-23-06848-s001.zip › ijms-1741587-supplementary/Supplementary_figures_files/Supp. Figures/FigureS4.pdf]
